# Ferrocene-Containing Sterically Hindered Phosphonium Salts

**DOI:** 10.3390/molecules23112773

**Published:** 2018-10-25

**Authors:** Vadim Ermolaev, Tatiana Gerasimova, Liliya Kadyrgulova, Ruslan Shekurov, Egor Dolengovski, Aleksandr Kononov, Vasily Miluykov, Oleg Sinyashin, Sergei Katsyuba, Yulia Budnikova, Mikhail Khrizanforov

**Affiliations:** Arbuzov Institute of Organic and Physical Chemistry, Kazan Scientific Center, Russian Academy of Sciences, 8 Arbuzov St., Kazan 420088, Russia; ermolaewadim@gmail.com (V.E.); tatyanagr@gmail.com (T.G.); liliya-kadyrgulova@mail.ru (L.K.); shekurovruslan@gmail.com (R.S.); cat_space@mail.ru (E.D.); kononovsnz97@gmail.com (A.K.); vasili.miluykov@mail.ru (V.M.); oleg@iopc.ru (O.S.); katsyuba@iopc.ru (S.K.); yulia@iopc.ru (Y.B.)

**Keywords:** ferrocene, phosphonium salts, ferrocene-containing compounds, DFT calculation, cyclic voltammetry, ionic liquids

## Abstract

The synthesis and physical properties of the series of the ferrocenyl-containing sterically hindered phosphonium salts based on di(*tert*-butyl)ferrocenylphosphine is reported. Analysis of voltamogramms of the obtained compounds revealed some correlations between their structures and electrochemical properties. The elongation of the alkyl chain at the P atom as well as replacement of the Br^−^ anion by [BF_4_]^−^ shifts the ferrocene/ferrocenium transition of the resulting salts into the positive region. DFT results shows that in the former case, the Br^−^ anion destabilizes the corresponding ion pair, making its oxidation easier due to increased highest occupied molecular orbital (HOMO) energy. Increased HOMO energy for ion pairs with the Br^−^ ion compared to BF_4_^−^ are caused by contribution of bromide atomic orbitals to the HOMO. The observed correlations can be used for fine-tuning the properties of the salts making them attractive for applications in multicomponent batteries and capacitors.

## 1. Introduction

**Figure 1:**
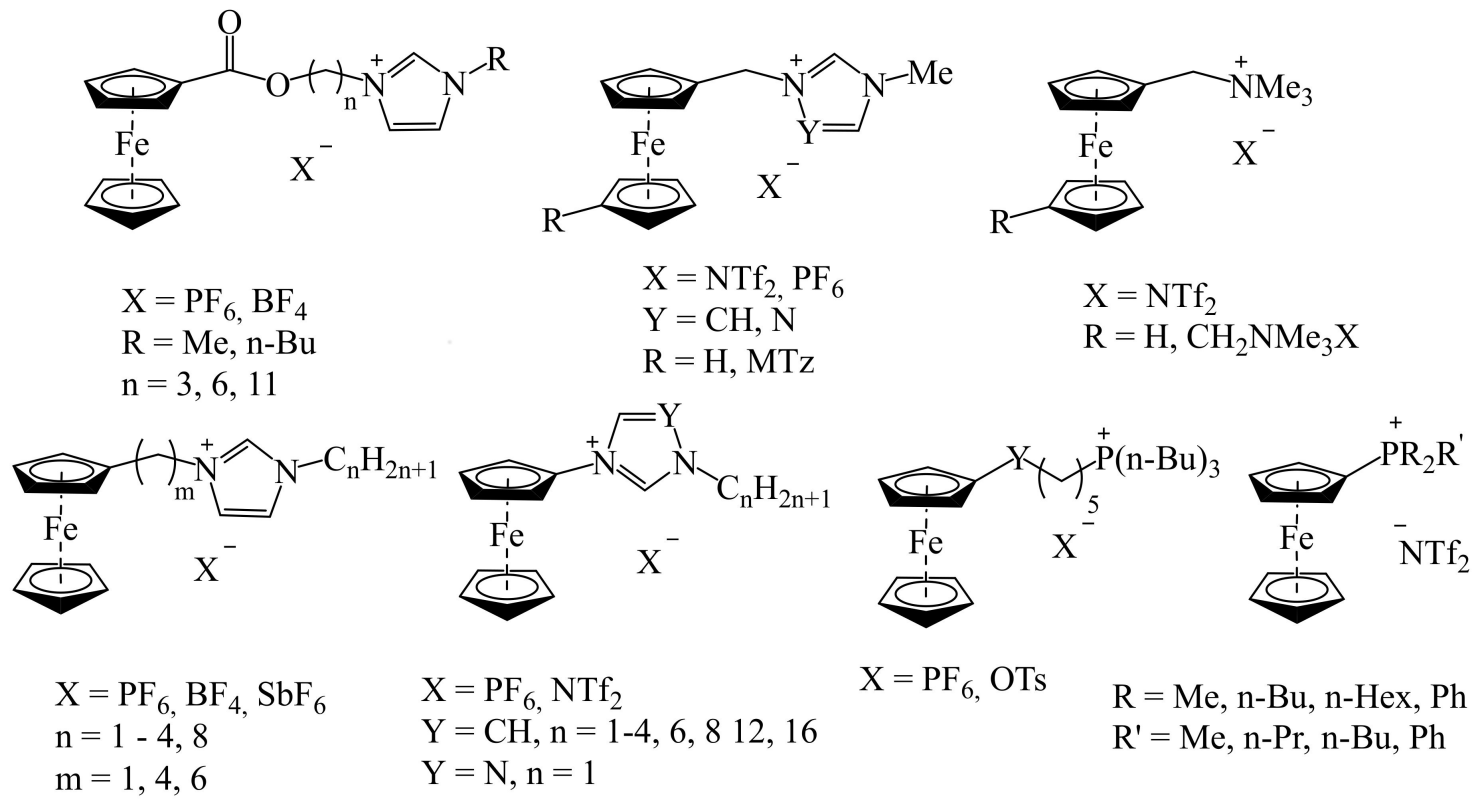
Ferrocenyl (Fc)-containing onium compounds [5,6,7,8,9,10].

Materials that enable selective ion transport under a wide range of conditions (temperature, pH) play a key role for chemical separation processes and electrochemical batteries [1,2]. One of the important ways to design such materials is development of synthetic strategies for producing sterically hindered phosphonium salts [2]. Phosphonium-based ionic liquids (ILs) are more thermally stable compared to nitrogen-containing analogues, which provides a wider range of applications (extended reaction temperature range, distillation of products, etc.) and decreases the amount of decomposition products in the reaction mixture [3]. In addition, the absence of an acidic proton, like in imidazolium salts, makes the ILs more stable under the reaction conditions [4]. Introduction of the ferrocenyl (Fc) fragment into ILs leads to unique electrochemical properties [5] (Figure 1), that allows the Fc-containing phosphonium salts to be used for modification of the platinum electrodes surfaces [6,7,8,9,10]. The latter also opens perspectives for using the Fc-based ILs as redox agents in electrolytes for lithium ion batteries [11]. This application requires careful choice of both cationic and anionic components. In this sense, [BF_4_]^−^ is attractive because it is one of the smallest and most available (from other anions, such as [PF_6_]^−^, [NTf]^−^, [SbF_6_]^−^, with comparable properties) weakly coordinating anion [12]. Moreover, potential batteries based on the [BF_4_]^−^ anion have excellent low-temperature characteristics [13]. In addition, this anion is electrochemically inert in a wide range of potentials [14].

**Figure 2:**
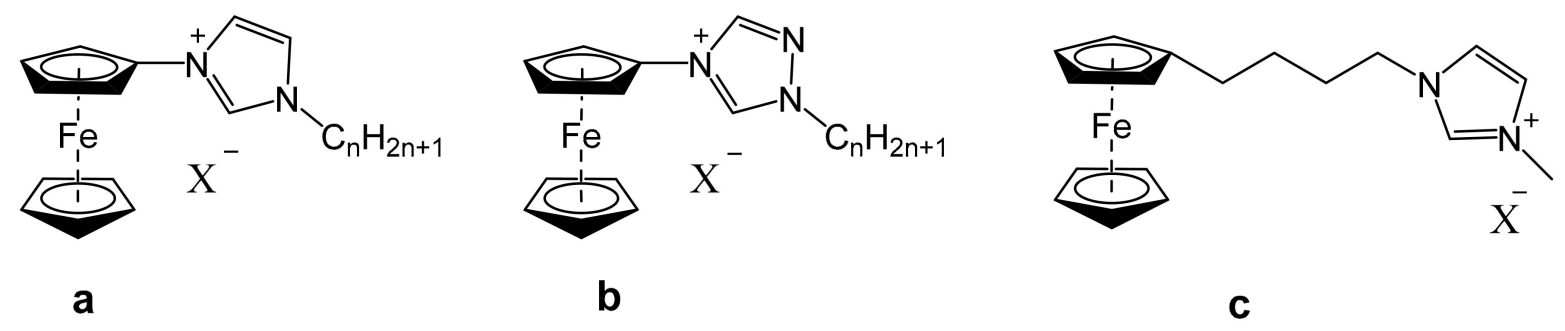
1-Ferrocenyl-3-alkylimidazolium (**a**); 1-ferrocenyl-4-methyltriazolium (**b**), and 1-(ferrocenylbutyl)-3-methylimidazolium (**c**) salts.

Among Fc-containing ILs, the best known and well-studied are the Fc-containing imidazolium salts (Figure 2). Inclusion of the Fc fragment to the imidazolium compounds increases melting points and viscosities. In these redox-active salts, Fc acts as a donor and an imidazolium cation as an acceptor [15]. The inclusion of an organometallic functional groups to the IL structure allows to change the physico-chemical properties of the salt, that enables their use in photoinduced electron transfer in solutions [9], and in the esterification reaction of aldehyde oxidation [16].

One of the main methods to study the redox properties of various organic compounds is voltammetry with chemically modified electrodes. Modified carbon-paste electrodes (CPE) are widely used due to their availability and simple manufacturing [17]. Nevertheless, the preparation of an optimal CPE with high conductivity, wide electrochemical window, and high stability and reproducibility, is still a challenge. Design of CPE requires using chemically inert compounds with high viscosity and ionic conductivity. Such electrodes can significantly increase the selectivity and sensitivity of voltammetric methods [18,19,20] and at the same time help to obtain important information about energy data [21,22,23].

A combination of the required properties makes phosphonium salts attractive for design of CPE. However, in the literature there are only a few examples of phosphonium salts used as a binder component for paste electrodes [17]. Thus, the aim of the work is to develop a synthetic strategy for obtaining the new Fc-containing phosphonium salts with a sterically hindered cation and to study their physico-chemical properties.

## 2. Results and Discussion

**Scheme 1:**
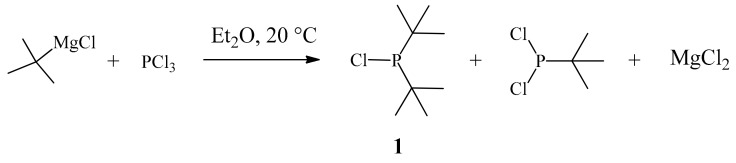
Preparation of di(*tert*-butyl)chlorophosphine.

Di(*tert*-butyl)ferrocenylphosphine has been prepared by the most common way, namely by lithiation of ferrocene with *tert*-butyllithium and subsequent reaction with di(*tert*-butyl)chlorophosphine **1** [23]. The starting di(*tert*-butyl)chlorophosphine has been obtained by the procedure described in [24], i.e., by the reaction of an excess of *tert*-butylmagnesium chloride with phosphorus trichloride with following separation of the product mixture by distillation under reduced pressure (Scheme 1).

**Scheme 2:**
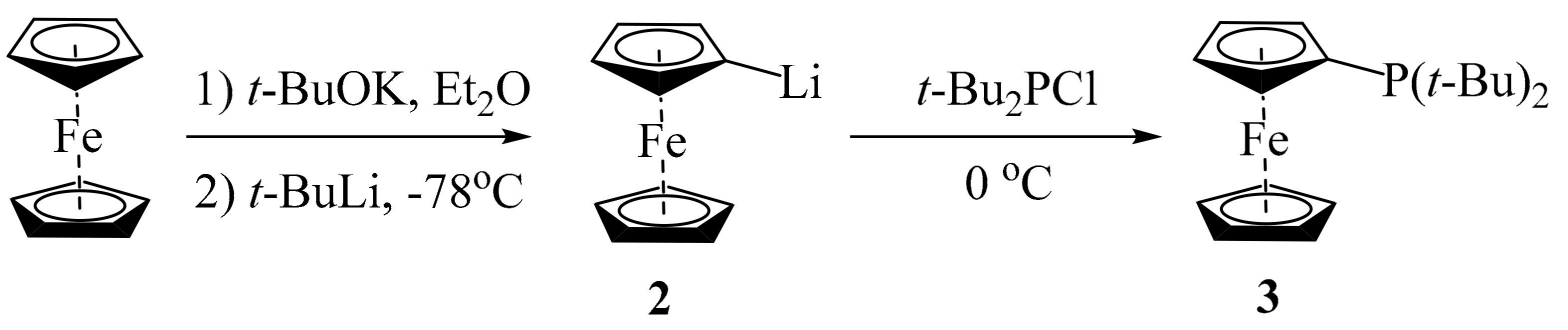
Preparation of di(*tert*-butyl)ferrocenylphosphine, **3**.

Lithiation with *tert*-butyllithium usually results in a mixture of mono- and 1,1-dilithiated derivatives. To avoid the formation of dilithiated compounds and obtain only monolithioferrocene, the reaction has been carried out in diethyl ether (Scheme 2).

Addition of *tert*-butyllithium to a solution of ferrocene and *t*-BuOK [25] in diethyl ether results in a color change of the solution from brown to orange, indicating that ferrocene was lithiated. Subsequent slow dropwise addition of di(*tert*-butyl)chlorophosphine **1** leads to precipitation of orange di(*tert*-butyl)ferrocenylphosphine **3**.

Phosphonium salts have been obtained by the reaction of di(*tert*-butyl)ferrocenylphosphine **3** with alkyl halides in an inert atmosphere (Table 1).

The temperature and time of the reaction are affected by the length of the alkyl group in the haloalkane. Reactions involving haloalkanes with a long alkyl chains proceeded at high temperatures and longer times. For example, the synthesis of methyl(di-*tert*-butyl)ferrocenylphosphonium iodide **4a** has been carried out at −70 °C, because of the strong exothermic effect, being completed in a few minutes. At the same time, the reaction of di(*tert*-butyl)ferrocenylphosphine with tetradecyl bromide was carried out at 100 °C for 6 h. Compared to well-known Fc-containing phosphines with *n*-alkyl substituents, sterically hindered analogs require slightly higher temperatures in the quaternization reaction with alkyl halides [5]. We performed the synthesis of phosphonium salts without any solvent. Under the reaction conditions, the reaction mixture is a viscous liquid. In the case of **4a**, the double excess of iodomethane acting as a solvent was used.

Synthesized Fc-containing phosphonium salts **4a**–**e** are soluble in dimethylsulfoxide (DMSO) and in methanol and a lower solubility is observed in chloroform. Non-polar solvents such as toluene, benzene, as well as esters and aliphatic solvents do not dissolve **4a**–**e**.

Fc-containing phosphonium salts **4a**–**e** are air-stable yellow-brown amorphous substances, however compound **4e** is hygroscopic. With the temperature increase, compounds **4a**–**e** gradually soften, become viscous, until they completely turn into liquids. The thermogravimetric/differential scanning calorimetry (TG-DSC) method shows that melting takes place in a rather wide temperature range for compounds **4a**–**d** (Table 1). The second peak is registered at a temperature of 165 °C (**4a**), 125 °C (**4b**), 141 °C (**4c**), 150 °C (**4d**), and 155 °C (**4e**), with a loss of mass. Most probably, this value corresponds to the decomposition temperature, as during preliminarily measurements of melting point at the Stuart smp30, the blackening of the samples was observed in this temperature range. Characteristic NMR shifts of synthesized salts are presented in Table 2.

**Figure 3:**
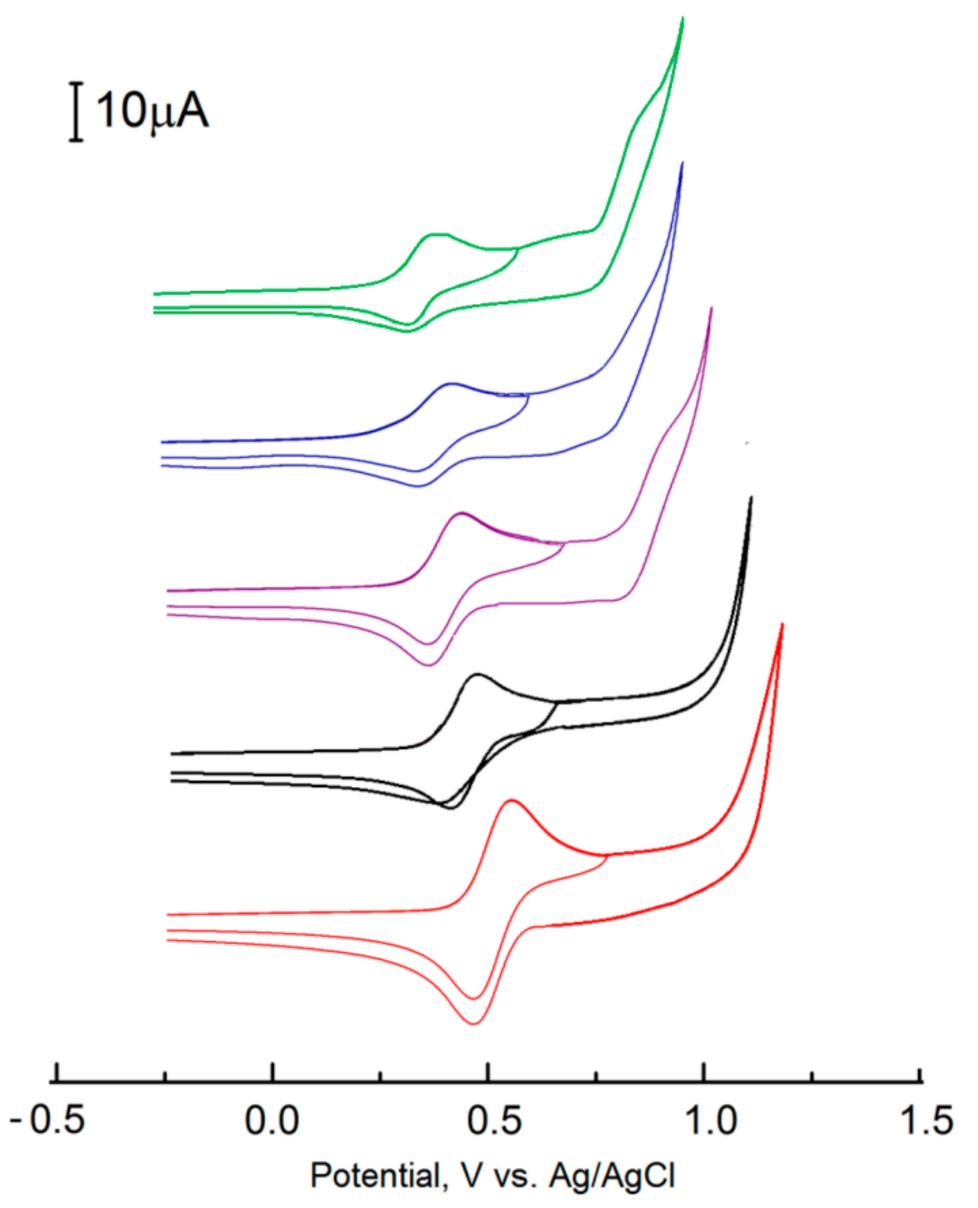
Cyclic voltammograms of **4a** (red), **4b** (green), **4c** (blue), **4d** (violet), and **4e** (black) oxidation and re-reduction (1 mM) in CH_3_CN, Bu_4_NBF_4_ (0.1M), RE: Ag/AgCl, scan rate 100 mV/s.

The electrochemical properties of **4a**–**e** in acetonitrile were studied by cyclic voltammetry (Figure 3).

In all cases, reversible transition of ferrocene⇿ferrocenium was observed in the anodic region. It should be noted, that for **4b**–**4d** with the same Br^−^ anion, increase of the length of the alkyl chain at the phosphorus atom is accompanied by the shift of the potential E_1/2_ (or E_semidiff_) to the anodic region (Table 3). This trend can be characterized by an increase in thermodynamic stability due to a decrease of HOMO energy. Compound **4a** with the I^−^ anion is oxidized more positively. It should be noted that the ferrocene fragment is HOMO energy probe ^1^E_HOMO_ = −(E_[semidif,ox vs. ferrocenium/ferrocene]_ + 4.8 V) [21].

At second oxidation potentials, irreversible oxidation of anionic fragments is observed [26]. In the cathode region (Table 3), an irreversible reduction of the phosphonium fragment is observed for all salts.

To expand the field of possible application of the obtained salts, the halide anion was replaced by a non-coordinating bulk tetrafluoroborate anion in the reaction of halides with a twofold excess of sodium tetrafluoroborate in ethyl alcohol (Table 4). The oxidation potential of the halide ions is close to 1 V, and the electrochemical window of the salts **4a**–**e** is 2.8–3.2 V (Table 3). Replacing the anion with a tetrafluoroborate ion was expected to increase these values.

The obtained phosphonium salts with tetrafluoroborate anion are dark brown substances, **5a**, **5c**, and **5e** are viscous, amorphous, and **5b** and **5d** are glassy. The substitution of the halide anion by the tetrafluoroborate anion results in a slight shift of the chemical shift (δP–CH_2_ in ^1^H NMR Spectra) to the weak fields (Table 5).

For all salts **5a**, **5c**–**e** electrospray ionization mass spectrometry (ESI) shows a peak at *m*/*z* = 87.2 in the region of negatively charged particles, that indicates a complete replacement of the bromide anion by a tetrafluoroborate anion.

For **5b** and **5c**, phase transitions have been registered by TG-DSC, decomposition was observed at 268 °C and 233 °C respectively. For compounds **5d** and **5e**, melting occurs in the temperature range 40–50 °C and 60–70 °C, respectively.

**Figure 4:**
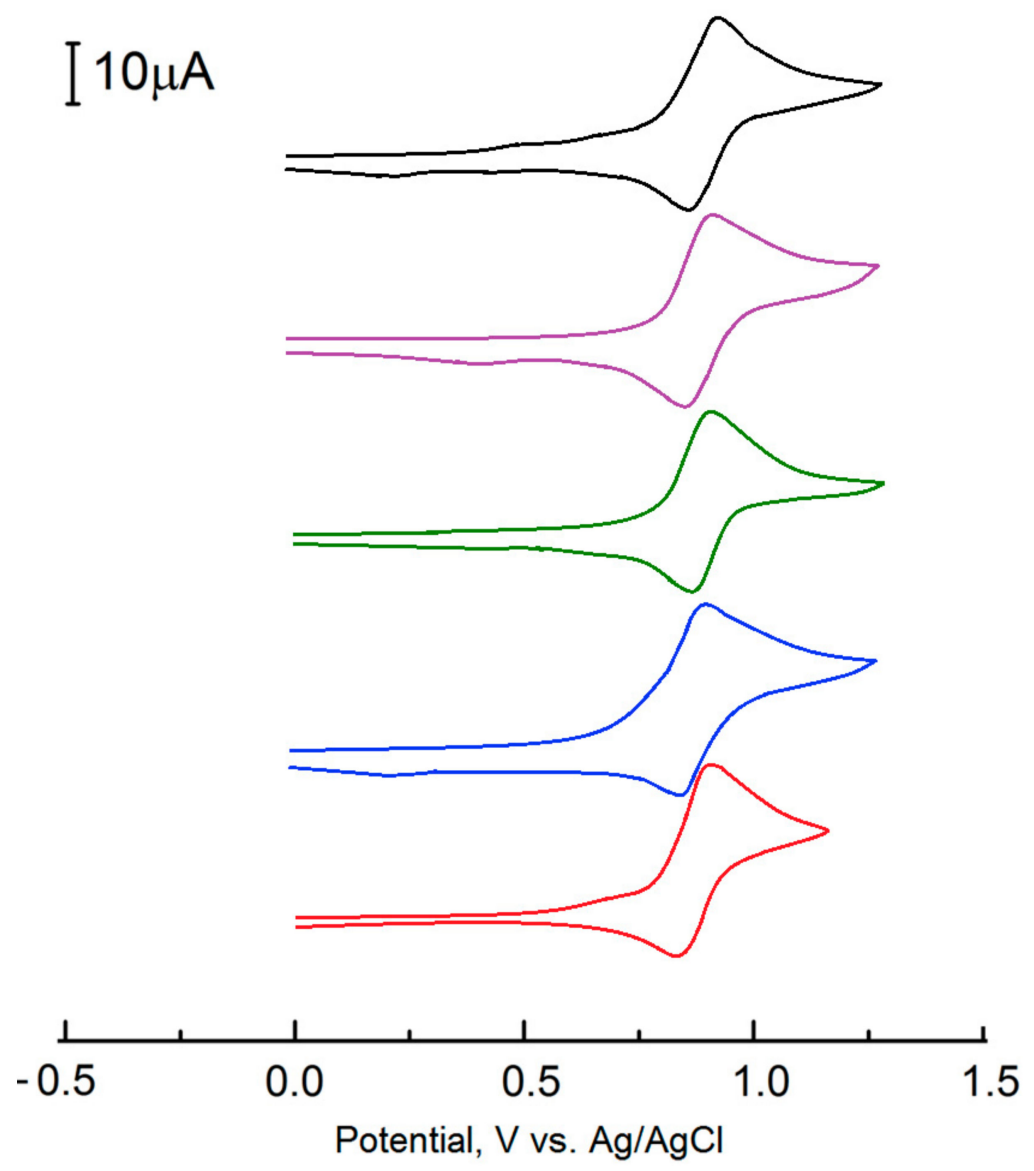
Cyclic voltammogram of **5a** (red), **5b** (blue), **5c** (green), **5d** (violet), **5e** (black) oxidation and re-reduction (1 mM) in CH_3_CN, Bu_4_NBF_4_ (0.1 M), reference electrode (RE) Ag/AgCl, scan rate 100 mV/s.

Analysis of cyclic voltammograms of **5a**–**e** in acetonitrile shows that the phosphonium salts with [BF_4_]^−^ anion (Figure 4, Table 6) are oxidized more positively compared to the Br^−^ salts (Figure 3, Table 3), which is also characterized by an increase in thermodynamic stability with an increase in the length of the alkyl tail at the phosphorus atom, potential E*_1/2_* (or E_semidiff_).

**Figure 5:**
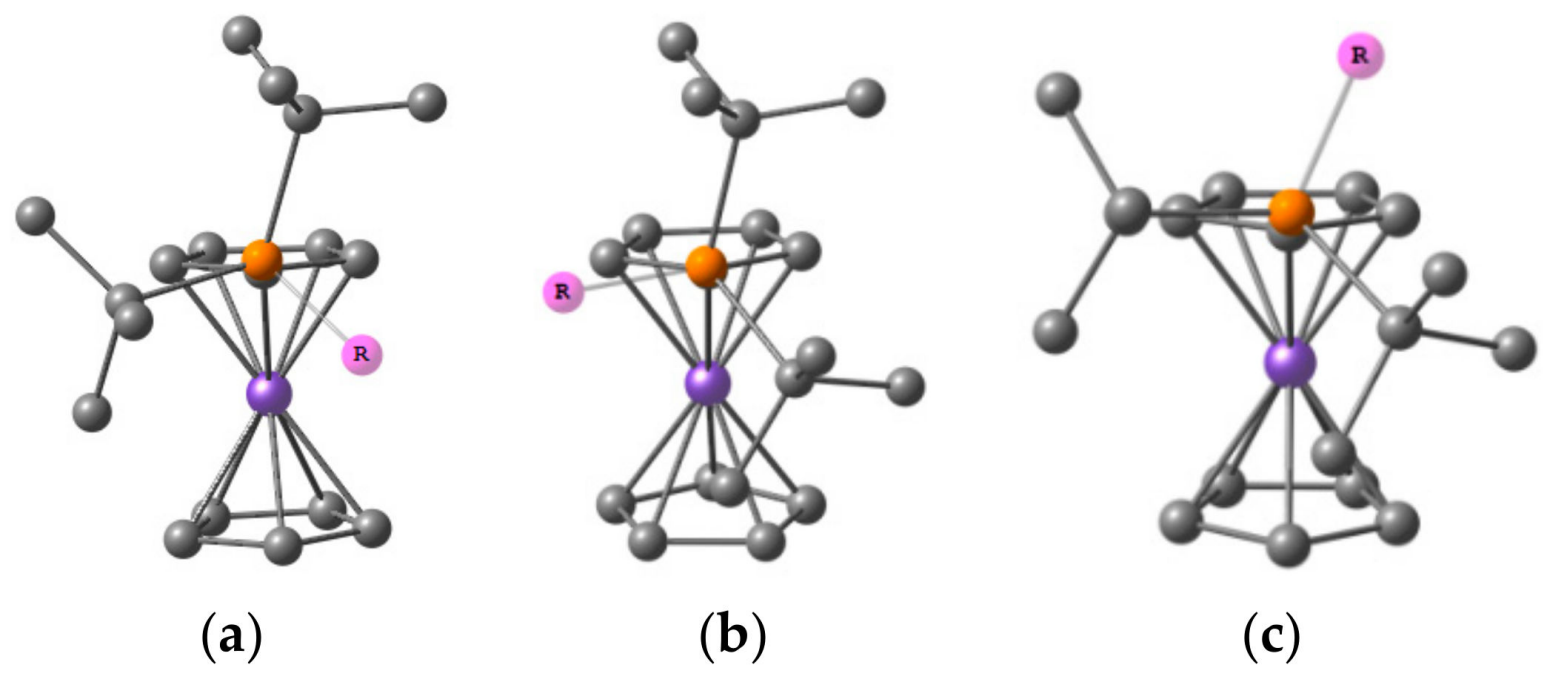
Considered conformations (**a**–**c**) of cation part of IL models, R = Me (**m1**), Et (**m2**), Pr (**m3**), Bu (**m4**), C_6_H_13_ (**m5**) C_7_H_15_ (**m6**), C_10_H_21_ (**m7**), C_13_H_27_ (**m8**), C_14_H_29_ (**m9**).

The series of ILs with different lengths of alkyl fragment at phosphorus atoms were considered quantum chemically (Figure 5). To simplify the computations, only the most stable all-trans conformations of alkyl moieties of [Fc-P(*t*-Bu)_2_Alk]^+^ cations were regarded. This planar zigzag conformation is preferred for the nearest to the phosphorus atom part of the alkyl chain in crystals of closely related phosphonium salts and is the most energetically stable form of isolated tetraalkylphosphonium cations according to quantum chemical computations [27]. For **m1**, **m5**, and **m9** models with the shortest, medium, and the longest alkyl chains, respectively, three possible conformations with different torsion (Cp)CCPC(Alk) angles were optimized (Figure 5). In **a** and **c** cases, quantum chemical optimization leads to structure with eclipsed Fc conformation, whereas in conformer **b**, the Fc moiety adopts a staggered conformation. The computed energy differences between **a** and **b** conformers with the alkyl moiety R turned towards the Fe^2+^ ion do not exceed 1 kcal/mol. The conformer **b** is the most stable form for the model **m1**, whereas for the models **m5** and **m9**, conformation **a** is energetically preferred. In all cases, the conformer **c** was predicted to be less energetically stable as compared to **a** and **b** by ~4 kcal/mol.

Energies of frontier molecular orbitals (FMOs) computed for **a** conformation of **m1**–**m9** models are collected in Table 7. According to our computations, elongation of the alkyl chain shifts both the highest occupied (HOMO) and the lowest unoccupied molecular orbitals (LUMO). This effect is slightly more pronounced for LUMO resulting in a weak increase of the HOMO–LUMO gap.

**Figure 6:**
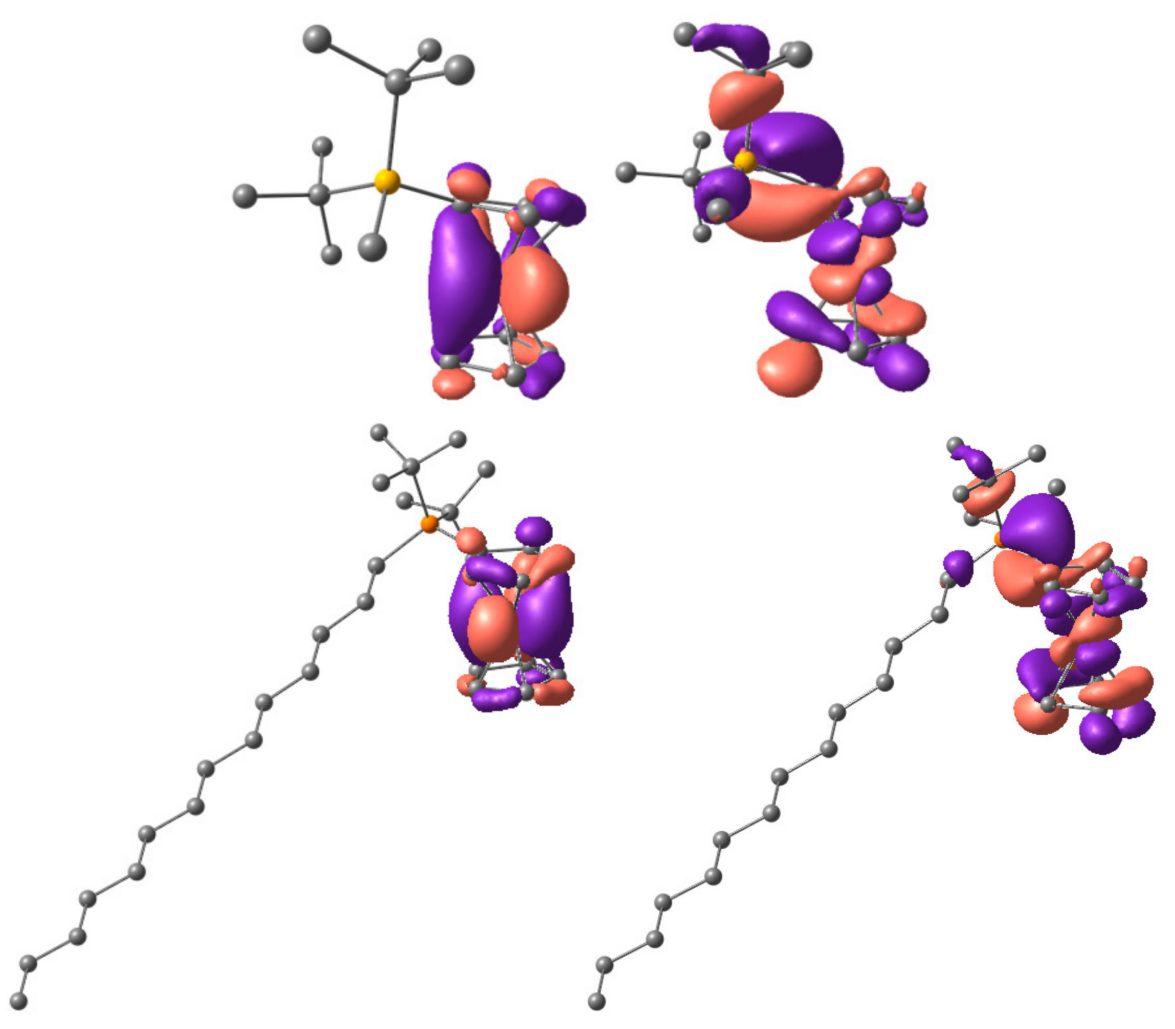
HOMO (**left**) and LUMO (**right**) of **m1** (**top**) and **m9** (**bottom**).

The computations showed similar composition of FMOs for m1 and m9. In both cases, HOMO is predicted to be localized on the Fc moiety of the cation, whereas LUMO is contributed also by P–C bond and atomic orbitals (AOs) of *t*-Bu and nearest carbon atom of alkyl group (Figure 6).

**Figure 7:**
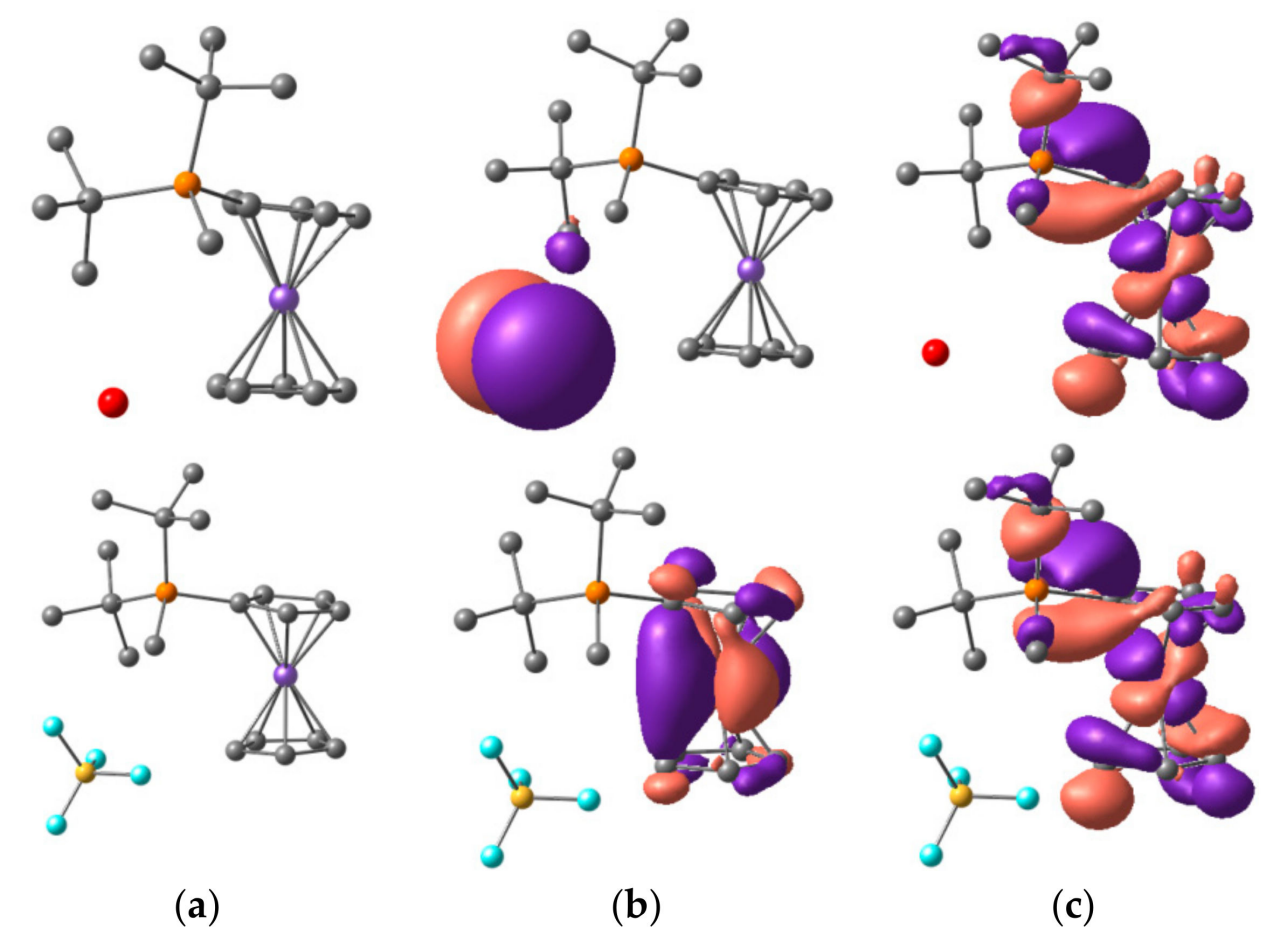
Structures of considered ion pairs of **m1** (**a**) with Br^−^ (**top**) and [BF_4_]^−^ (**bottom**) anions and corresponding HOMO (**b**) and LUMO (**c**).

For **m1** and **m9**, ion pairs with Br^−^ and [BF_4_]^−^ counterions were optimized (Figure 7). According to our computations, the energy of ion pairing is higher by 6–8 kcal/mol for Br^−^ anion compared to [BF_4_]^−^ (84 vs. 78 kcal/mol for Fc-P^+^(*t*-Bu)_2_CH_3_ and 85 vs. 77 for Fc-P^+^(*t*-Bu)_2_C_14_H_29_ kcal/mol).

The ion pairing increases the energies of both HOMO and LUMO; in the case of [BF_4_]^−^ anion, the shift is almost parallel, being slightly more pronounced for LUMO, resulting in quite modest increasing of the HOMO–LUMO gap compared to cations. In contrast, Br^−^ much more essentially affects the HOMO, making the oxidation of bromide salts easier. The resulting HOMO–LUMO gap is significantly decreased compared to cations and bromides (Table 7). Such pronounced effect in the latter case is caused by contribution of Br^−^ AOs to HOMO (Figure 5). Similar localization of HOMO and LUMO is predicted also for **m9** with [BF_4_]^−^ and Br^−^ anions. In both cases, elongation of alkyl chain of cationic moiety leads to reduced HOMO energy (from −4.93 to −5.01 in the Br^−^ case and from −6.61 to −6.67 for [BF_4_]^−^), that is in accordance with the experimental trends observed electrochemically. Indeed, according to the abovementioned equation ^1^E_HOMO_ =−(E_[semidif,ox vs. ferrocenium/ferrocene]_ + 4.8 V) the latter is equal to −5.23 for Fc-P^+^(*t*-Bu)_2_C_3_H_7_Br^−^ and −5.26 for Fc-P^+^(*t*-Bu)_2_C_14_H_29_Br^−^; −5.69 for Fc-P^+^(*t*-Bu)_2_CH_3_BF_4_^−^ and −5.71 for Fc-P^+^(*t*-Bu)_2_C_14_H_29_BF_4_^−^.

## 3. Materials and Methods

### 3.1. Electrochemistry

Cyclic voltammograms were recorded with a BASi Epsilon E2P (Bioanalytical Systems, Inc., West Lafayette, IN, USA) potentiostat. The device comprises a measuring unit, PC Dell Optiplex 320 (Bioanalytical Systems, Inc.) with the Epsilon-EC-USB-V200 software (Bioanalytical Systems, Inc.). Tetrabutylammonium tetrafluoroborate (C_4_H_8_)_4_NBF_4_ was used as background electrolyte. The working electrode was a stationary disc glassy-carbon electrode (the surface area of 6 mm^2^). Ag/AgCl (0.01 M KCl) (Bioanalytical Systems, Inc.) was used as a reference electrode. The reference electrode was connected with the cell solution by a modified Luggin capillary filled with the supporting electrolyte solution (0.1 M Bu_4_NBF_4_ in CH_3_CN). Thus, the reference electrode assembly had two compartments, each terminated with an ultra-fine glass frit to separate the AgCl from the analyte. A platinum wire was used as an auxiliary electrode. The scan rate was 100 mV/s. The measurements were performed in a temperature-controlled electrochemical cell (volume from 1 mL to 5 mL) in an inert gas atmosphere (N_2_). Between measurements or prior to a registration of a voltammetry wave, the solution was actively stirred with a magnetic stirrer under constant inflow of an inert gas that was run through a dehydrating system, and then through a nickel-based purification system BI-GAS cleaner (OOO Modern Laboratory Equipment, Novosibirsk, Russia) to remove trace quantities of oxygen.

### 3.2. NMR Experiments

The NMR spectra were registered on the equipment of Assigned Spectral-Analytical Center of FRC Kazan Scientific Center of RAS, namely on multi-nuclear spectrometer Bruker AVANCE-400 (BRUKER BioSpin, Rheinstetten, Germany) (400.1 MHz (^1^H), 100.6 MHz (^13^C) and 162.0 MHz (^31^P)). Chemical shifts are given in parts per million relative to SiMe_4_ (^1^H, internal) and 85% H_3_PO_4_ (^31^P, external).

### 3.3. Thermogravimetric/Differential Scanning Calorimetry (TG-DSC)

Thermogravimetric analysis was performed on the NETZSCH STA 449F3 (NETZSCH-Gerätebau GmbH, Selb, Germany) with a heating rate of 10 K per minute up to 400 °C under argon atmosphere.

### 3.4. Calculations

All calculations were performed with the Gaussian 16 suite of programs [28]. The hybrid PBE0 functional [29] and the Ahlrichs’ triple-ζ def-TZVP AO basis set [30] were used for optimization of all structures. In all geometry optimizations, the D3 approach [31] to describe the London dispersion interactions together with the Becke–Johnson (BJ) damping function [32,33,34] were employed as implemented in the Gaussian 16 program.

### 3.5. Mass-Spectra

Mass Electrospray ionization mass spectrometry (ESI-MS) was performed on the AmazonX mass spectrometer (Bruker Daltonics, Bremen, Germany). The measurements were carried out in the positive/negative ion detection mode in the *m/z* range from 100 to 1000. The voltage on the capillary was 140 V. Data was processed using the DataAnalysis 4.0 program (Bruker Daltonics).

### 3.6. Materials and Reagents

All the work related to the preparation of the initial reagents, the synthesis and the release of products was carried out in an inert atmosphere using standard Schlenk apparatus. All solvents and purchased reagents were absolute by the appropriate methods, mainly by distillation in an inert atmosphere.

*t*-BuLi (1.6 M solution) (Sigma Aldrich, St. Louis, MO, USA) was used without a preliminary purification.

### 3.7. Synthesis (General Procedure)

#### 3.7.1. Synthesis of Di(*tert*-Butyl)Ferrocenylphosphine

In a two-necked Schlenk vessel equipped with a magnetic stirrer, ferrocene (10.3 mol, 1.923 g) and *t*-BuOK (1.55 mmol, 0.173 g) were placed, the mixture was dissolved in 150 mL of diethyl ether. The solution was cooled to −78 °C. A solution of *t*-BuLi (10.3 mmol) was added over 10–15 min. Stirring of the reaction mixture was carried out at −70 °C for 1 h. An orange precipitate of di(*tert*-butyl)ferrocenylphosphine was observed to form. The reaction mixture then was warmed to 0 °C and (*t-*Bu)_2_PCl (5.15 mmol, 0.98 mL) was added dropwise. After the completion of the dropping, the cooling bath was removed and the reaction mixture was warmed to room temperature. After Et_2_O was removed in vacuum theresidue was dissolved in 200 mL of petroleum ether and filtered through a short silica column. Removal of the solvents yield a dark-brown solid 2.431 g (71%). ^31^P (Petroleum ether, δ, ppm) NMR: 28.5 (s). [5]

#### 3.7.2. Method for the Synthesis of Methyl (Di-*tert*-butyl)Ferrocenylphosphonium Iodide (**4a**)

Iodomethane (0.191 mL, 3.06 mmol) was added to di(*tert*-butyl)ferrocenylphosphine (0.507 g, 1.53 mmol) with stirring. The precipitation of a brown solid was observed. The product is then washed 2 times with 20 mL of petroleum ether and 2 times with 20 mL of diethyl ether. The precipitate is filtered out, the solvent residues are removed in vacuo. Yield 0.449 g (62%), m.p. = 74.1 °C.

#### 3.7.3. Synthesis of Propyl-, Hexyl-, Decyl-, Tetradecyl (Di-*tert*-butyl)ferrocenylphosphonium Bromides (**4b**–**e**)

An equivalent amount of alkyl bromide was added to di(*tert*-butyl)ferrocenylphosphine. The reaction mass was stirred at 70 °C (**4b**), 75 °C (**4c**), 90 °C (**4d**), and 100 °C (**4e**) for 5 h. The product was then washed with 2 portions of 20 mL of petroleum ether and 2 portions of 20 mL of diethyl ether. The precipitate was filtered out and the solvent residues were removed in vacuo.

#### 3.7.4. Synthesis of Methyl-, Propyl-, Hexyl-, Decyl-, Tetradecyl-(di-*tert*-butyl)ferrocenylphosphonium Tetrafluoroborates (**5a**–**e**)

Salts (**5a**–**e**) were dissolved in 10 mL of ethyl alcohol and a twofold excess of sodium tetrafluoroborate solution in ethyl alcohol was added to them. The reaction mixture was stirred for 12 h. The solvent was evaporated in vacuo, and the salt was dissolved in 20 mL of methylene chloride and washed with distilled water. The organic phase was separated and dried over magnesium sulfate. The solvent is evaporated, and the salt is dried under vacuum at 50 °C for 8 h. For other details, see the supplementary material.

## 4. Conclusions

A synthetic methodology for the preparation of new Fc-containing sterically hindered phosphonium ILs based on di(*tert*-butyl)ferrocenylphosphine is described. Electrochemical measurements showed that with increasing length of the alkyl fragment at the phosphorus atom, the potential shifts to the anode region. The replacement of the bromide anion by [BF_4_]^−^ significantly shifts the ferrocene/ferrocenium transition into the positive region of the resulting salts. Such a pronounced effect is caused by a stronger ion pairing with a Br^−^ anion. According to our computations, in the ion pairs with Br^−^, the HOMOs are contributed by the counter-ion. For both Br^−^ and [BF_4_]^−^ cases, elongation of the alkyl chain with a cationic moiety leads to a reduction of HOMO energy that is in accordance with the experimental trends observed electrochemically.

The electrochemical properties of the newly synthesized Fc-containing phosphonium salts mean that they will represent attractive alternative components for batteries and capacitors. This opens additional potential for using Fc-based ILs as redox agents in electrolytes for lithium ion batteries.

## Figures and Tables

**Table 1:**
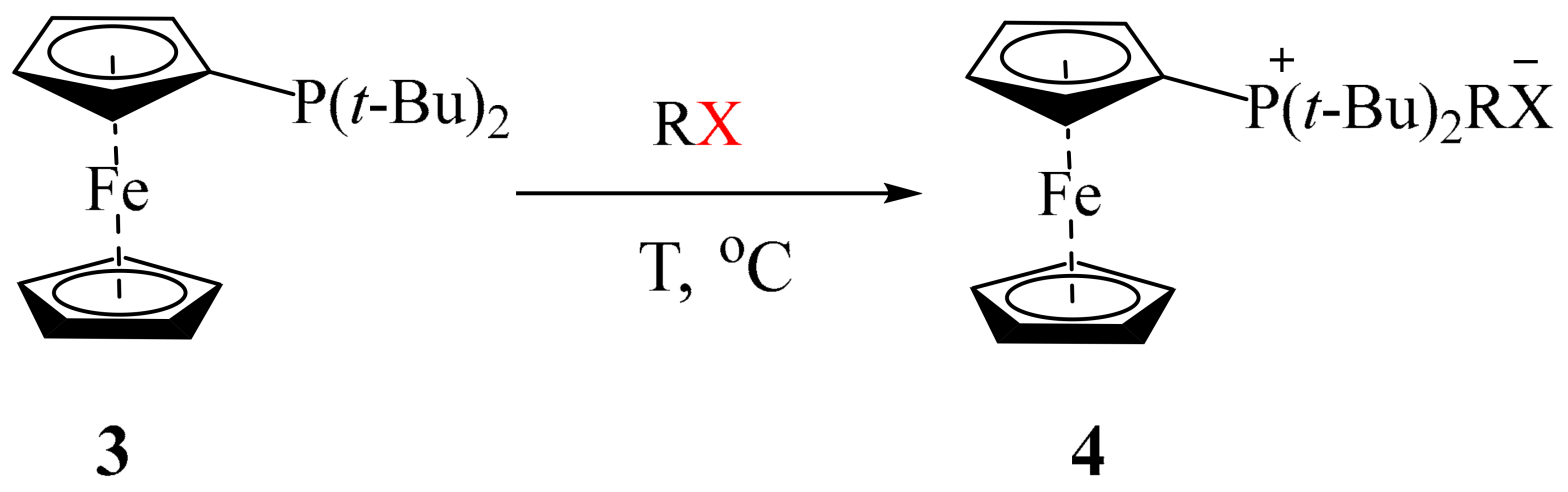
Preparation of ferrocene-containing phosphonium salts. Details of the reaction conditions are in the supplementary material.

No.	RX	T (°C)	Reaction Time (h)	Yield (%)
**4a**	CH_3_I	−70	0.5	62
**4b**	n-C_3_H_7_Br	70	5	78
**4c**	n-C_6_H_13_Br	75	5	71
**4d**	n-C_10_H_21_Br	90	6	69
**4e**	n-C_14_H_29_Br	100	8	59

**Table 2:** ^1^H and ^31^P chemical shifts of Fc-containing phosphonium salts **4a**–**e**^a^.

No.	Phosphonium Salts	δP–CH_2_ in ^1^H NMR Spectra	δ ^31^P NMR Spectra	Temperature Range of Melting (°C)	Decomposition Temperature (°C)
**4a**	Fc-P^+^(*t*-Bu)_2_CH_3_I^−^	2.43	48.34	69–80	165
**4b**	Fc-P^+^(*t*-Bu)_2_C_3_H_7_Br^−^	2.91	48.59	60–70	125
**4c**	Fc-P^+^(*t*-Bu)_2_C_6_H_13_Br^−^	2.47	47.85	57–68	141
**4d**	Fc-P^+^(*t*-Bu)_2_C_10_H_21_Br^−^	2.43	47.45	58–70	150
**4e**	Fc-P^+^(*t*-Bu)_2_C_14_H_29_Br^−^	2.58	47.76	61–71	155

^a^ Fc = Fe(η^5^-C_5_H_5_)(η^5^-C_5_H_4_).

**Table 3:** Electrochemical properties of Fc-containing phosphonium salts **4a**–**4e**. Conditions: Room temperature, Working Electrode: Glass-carbon, vs. Ag/AgCl. Solvent CH_3_CN.

No.	Phosphonium Salts	^1^ E_semidiff_^ox^ (V)	^2^ E_onset_^ox^ (V)	^1^ E_onset_^red^ (V)
**4a**	Fc-P^+^(*t*-Bu)_2_CH_3_I	0.50	1.10	−1.70
**4b**	Fc-P^+^(*t*-Bu)_2_C_3_H_7_Br^−^	0.43	0.80	−2.09
**4c**	Fc-P^+^(*t*-Bu)_2_C_6_H_13_Br^−^	0.44	0.80	−2.07
**4d**	Fc-P^+^(*t*-Bu)_2_C_10_H_21_Br^−^	0.45	0.84	−2.06
**4e**	Fc-P^+^(*t*-Bu)_2_C_14_H_29_Br^−^	0.46	1.16	−2.05

^a^ The table shows the averaged shifts made after 20 measurements. Fc = Fe(η^5^-C_5_H_5_)(η^5^-C_5_H_4_). E_1/2_ of ferrocene at the same conditions is equal to 0.42 V.

**Table 4:**
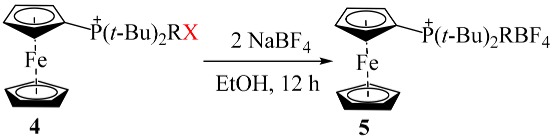
Reaction of substitution of anions in Fc-containing phosphonium salts.

No.	RX	Yield (%)
**5a**	CH_3_I	86
**5b**	n-C_3_H_7_Br	82
**5c**	n-C_6_H_13_Br	81
**5d**	n-C_10_H_21_Br	89
**5e**	n-C_14_H_29_Br	81

**Table 5:** ^1^H and ^31^P chemical shifts of Fc-containing phosphonium salts **5a**–**e**^a^.

No.	Phosphonium Salts	δP–CH_2_ in ^1^H NMR Spectra	δ ^31^P NMR Spectra	Temperature Range of Melting (°C)	Decomposition Temperature (°C)
**5a**	Fc-P^+^(*t*-Bu)_2_CH_3_BF_4_^−^	2.18	49.54	69–80	164
**5b**	Fc-P^+^(*t*-Bu)_2_C_3_H_7_BF_4_^−^	2.51	47.44	39–45	275
**5c**	Fc-P^+^(*t*-Bu)_2_C_6_H_13_BF_4_^−^	2.52	48.11	51–52	257
**5d**	Fc-P^+^(*t*-Bu)_2_C_10_H_21_BF_4_^−^	2.56	48.32	34–38	227
**5e**	Fc-P^+^(*t*-Bu)_2_C_14_H_29_BF_4_^−^	2.57	48.54	57–60	245

^a^ Fc = Fe(η^5^-C_5_H_5_)(η^5^-C_5_H_4_).

**Table 6:** Electrochemical properties of ferrocene-containing phosphonium salts. Conditions: room temperature, working electrode: glass-carbon, RE: Ag/AgCl, solvent: CH_3_CN ^a^.

No.	Phosphonium Salts	^1^ E_semidiff_^ox^ (V)	^2^ E_onset_^ox^ (V)	^1^ E_onset_^red^ (V)
**5a**	Fc-P^+^(*t*-Bu)_2_CH_3_BF_4_^−^	0.89	2.95	−1.82
**5b**	Fc-P^+^(*t*-Bu)_2_C_3_H_7_BF_4_^−^	0.89	2.95	−1.94
**5c**	Fc-P^+^(*t*-Bu)_2_C_6_H_13_BF_4_^−^	0.89	2.95	−2.05
**5d**	Fc-P^+^(*t*-Bu)_2_C_10_H_21_BF_4_^−^	0.90	2.96	−2.20
**5e**	Fc-P^+^(*t*-Bu)_2_C_14_H_29_BF_4_^−^	0.91	2.97	−2.40

^a^ The table shows the averaged shifts made after 20 measurements. ^a^ Fc = Fe(η^5^-C_5_H_5_)(η^5^-C_5_H_4_).

**Table 7:** Absolute HOMO and LUMO energies and HOMO–LUMO gaps (in eV) calculated for the models **m1**–**m9** ^a^.

Compound	HOMO	LUMO	LUMO–HOMO Gap
Fc-P^+^(*t*-Bu)_2_CH_3_ (**m1**)	−9.24	−3.58	5.67
Fc-P^+^(*t*-Bu)_2_CH_3_BF_4_^−^	−6.61	−0.91	5.70
Fc-P^+^(*t*-Bu)_2_CH_3_Br^−^	−4.93	−0.74	4.19
Fc-P^+^(*t*-Bu)_2_C_2_H_5_ (**m2**)	−9.24	−3.56	5.68
Fc-P^+^(*t*-Bu)_2_C_3_H_7_ (**m3**)	−9.22	−3.53	5.69
Fc-P^+^(*t*-Bu)_2_C_4_H_8_ (**m4**)	−9.20	−3.51	5.69
Fc-P^+^(*t*-Bu)_2_C_6_H_13_ (**m5**)	−9.18	−3.49	5.69
Fc-P^+^(*t*-Bu)_2_C_7_H_15_ (**m6**)	−9.18	−3.49	5.69
Fc-P^+^(*t*-Bu)_2_C_10_H_21_ (**m7**)	−9.17	−3.48	5.69
Fc-P^+^(*t*-Bu)_2_C_13_H_27_ (**m8**)	−9.16	−3.48	5.69
Fc-P^+^(*t*-Bu)_2_C_14_H_29_ (**m9**)	−9.16	−3.47	5.69
Fc-P^+^(*t*-Bu)_2_C_14_H_29_BF_4_^−^	−6.67	−0.96	5.71
Fc-P^+^(*t*-Bu)_2_C_14_H_29_Br^−^	−5.01	−0.73	4.28

^a^ Fc = Fe(η^5^-C_5_H_5_)(η^5^-C_5_H_4_).

## Supplementary Materials

The following are available online: Experimental procedures; NMR spectra of synthesized compounds; ESI-MS spectra of synthesized compounds and TG-DSC curves of synthesized compounds.
