# Supplementary material for: Ferrocene-Containing Sterically Hindered Phosphonium Salts
Source: Molecules. 2018 Oct 25;23(11):2773. doi: 10.3390/molecules23112773 (PMC6278326; doi:10.3390/molecules23112773)
Supplement: Supplementary file 1 [file molecules-23-02773-s001.pdf]

Supporting Information for

Ferrocene-Containing Sterically Hindered Phosphonium Salts

Vadim Ermolaev, Tatiana Gerasimova, Liliya Kadyrgulova, Ruslan Shekurov, Egor Dolengovski, Aleksandr Kononov, Vasily Miluykov, Oleg Sinyashin, Sergei Katsyuba, Yulia Budnikova and Mikhail Khrizanforov*

[a] Arbuzov Institute of Organic and Physical Chemistry of FRC Kazan Scientific Center of RAS,
Russian Academy of Sciences, 8 Arbuzov St., Kazan 420088, Russian Federation
∗Corresponding author. Tel.: +7 9625612219; fax: +78432732253
E-mail address: khrizanforov@iopc.ru, khrizanforov@gmail.com

Experimental

1. Electrochemistry

Cyclic voltammograms were recorded with a BASi Epsilon E2P (USA) potentiostat. The device comprises a measuring unit, PC DellOptiplex 320 with the Epsilon-EC-USB-V200 software. Tetrabutylammonium tetrafluoroborate $(C_4H_8)_4NBF_4$ was used as background electrolytes. The working electrode was a stationary disc glassy-carbon electrode (the surface area of 6 mm^2). Ag/AgCl (0.01 M KCl) was used as a reference electrode. The reference electrode was connected with the cell solution by a modified Luggin capillary filled with the supporting electrolyte solution (0.1 M Bu_4NBF_4 in CH_3CN). Thus, the reference electrode assembly had two compartments, each terminated with an ultra-fine glass frit to separate the AgCl from the analyte. A platinum wire was used as an auxiliary electrode. The scan rate was 100 $mV \cdot s^{-1}$. The measurements were performed in a temperature-controlled electrochemical cell (volume from 1 mL to 5 mL) in an inert gas atmosphere (N_2). Between measurements or prior to a registration of a voltammetry wave, the solution was actively stirred with a magnetic stirrer in the atmosphere of constant inflow of an inert gas that was run through a dehydrating system, and then through a nickel-based purification system BI-GAS cleaner (manufactured by OOO Modern Laboratory Equipment, Novosibirsk) to remove trace quantities of oxygen.

2. NMR experiments

NMR spectra were recorded with multi-nuclear spectrometer Bruker AVANCE-400 (400.1 MHz (^{1}H), 100.6 MHz (^{13}C) and 162.0 MHz (^{31}P)). Chemical shifts are given in parts per million relative to $SiMe_4$ (1H, internal solvent) and 85% H_3PO_4 (^{31}P, external).

3. TG-DSC and melting point measurement

Thermogravimetric analysis was performed on the NETZSCH STA 449F3 with a heating rate 10 K per minute up to 400°C in an argon atmosphere. Melting points was measured with Stuart SMP30 (Stuart, Cole-Parmer, UK).

4. Calculation

All calculations were performed with the Gaussian 16 suite of programs [1] The hybrid PBE0 functional [2] and the Ahlrichs' triple-ζ def-TZVP AO basis set [3] were used for optimization of all structures. In all geometry optimizations the D3 approach [4] to describe the London dispersion interactions together with the Becke–Johnson (BJ) damping function [5-7] were employed as implemented in the Gaussian 16 program.

5. Mass Electrospray ionization mass spectrometry (ESI-MS) was performedon the AmazonX mass spectrometer (Bruker Daltonik GmbH, Bremen, Germany). The measurements were carried out in the positive/negative ion detection mode in the m/z range from 100 to 1000. The voltage on the capillary was 140 V. Data was processed using the DataAnalysis 4.0 program (Bruker Daltonik GmbH, Bremen, Germany)

6. Reagents and research subjects

All the work related to the preparation of the initial reagents, the synthesis and the release of products, was carried out in an inert atmosphere using standard Schlenk apparatus. All solvents and purchased reagents were absolute by the appropriate methods, mainly by distillation in an inert atmosphere.

t-BuLi ("1.6M solution") (by Sigma Aldrich) were used without a preliminary purification.

7. Synthesis

7.1 General procedure

The procedure for the synthesis of di(*tert*-butyl)ferrocenylphosphine In a two-necked Schlenk vessel equipped with a magnetic stirrer, ferrocene (10.3 mol, 1.923 g) and t-BuOK (1.55 mmol, 0.173 g) were placed, the mixture was dissolved in 150 ml diethyl ether. The solution was cooled to -78 ° C. A solution of t-BuLi (10.3 mmol) was added over 10-15 minutes. Stirring of the reaction mixture was carried out at -70 ° C. for 1 hour. An orange precipitate of di(*tert*-butyl) ferrocenylphosphine was observed to form. The reaction mixture then was warmed to 0 °C and *t*-Bu_2PCl (5.15 mmol, 0.98 mL) dropwise was added. After the completion of the dropping, the cooling bath was removed, the reaction mixture was warmed to room temperature. After Et_2O was removed in vacuum, residue was dissolved in 200 ml of the petroleum ether and filtered through short silica column. Remove of the solvents yields dark-brown solid 2.431 g (71%). ^{31}P (Petroleum ether, δ, ppm) NMR: 28.5 (s).

Method for the synthesis of methyl(di-*tert*-butyl)ferrocenylphosphonium iodide (4a).
Iodomethane (0.191 ml, 3.06 mmol) was added to di(*tert*-butyl)ferrocenylphosphine (0.507 g, 1.53 mmol) with stirring at -70°C for 30 min. After that reaction mixture was left to warm up to room temperature. The product was then vigorously stirred twice with 20 ml of petroleum ether and twice with 20 ml of diethyl ether in 10 minutes each. After each stirring, the solvent was removed with a cannula with a filter nozzle, the solvent residues are removed in vacuo. Yield 0.449 g (62%). Mp = 74° C. ^{31}P NMR (162 MHz, $CDCl_3$, δ) 48.34; ^{1}H NMR (400 MHz, $CDCl_3$, δ), 1.49 (d, $^3J_{PH}$ = 15.47 Hz, 18H), 2.43 (d, $^2J_{PH}$ = 10.09, 3H), 4.46 (s, 5H), 4.74 (s, 2H), 4.83(s, 2H); ^{13}C{1H} NMR (100 MHz, $CDCl_3$, δ) 18.98 (d, $^1J_{PC}$ = 36.83 Hz), 27.24, 30.78 (m), 60.5 (d, $^1J_{PC}$ = 86.03 Hz), 71.13, 72.38 (d, $^2J_{PC}$ = 8.38 Hz), 73.22(d, $^3J_{PC}$ = 9.90 Hz) 345.2 $[M]^+$. Anal. calc. (%):C, 48.33; H, 6.40; Fe, 11.83; I, 26.88; P, 6.56. Found: C, 48.41; H, 6.35; I, 27.22; Fe, 11.65; P, 6.37.

General procedure for the synthesis of propyl-, hexyl-, decyl-, tetradecyl (di-*tert*-butyl)ferrocenylphosphonium bromides (**4b-e**). An equivalent amount of alkyl bromide was added to di-*tert*-butylferrocenylphosphine. The reaction mass was stirred at 70°C. (**4b**), 75°C. (**4c**), 90°C. (**4d**) and 100°C (**4e**) for 5 hours. The product was vigorously stirred with 2 portions of 20 ml of petroleum ether and 2 portions of 20 ml of diethyl ether. After each stirring, the solvent was removed with a cannula with a filter nozzle, the solvent residues are removed in vacuo.

Propyl(di-*tert*-butyl)ferrocenylphosphonium bromide **(4b)**.
Orange solid, yield 0.426 g (78 %), m.p. 60-70 °C. ^{31}P NMR (162 MHz, $CDCl_3$, δ) 48.57; ^{1}H NMR (400 MHz, $CDCl_3$, δ) 1.29 (m, 3H, CH_3), 1.53 (d, $^3J_{PH}$ = 14.17 Hz, 18H), 2.18 (m, 2H), 2.91 (m, 2H), 4.43 (s, 5H), 4.78 (s, 2H), 4.85 (s, 2H, H_{Cp}). ^{13}C{1H} NMR (100 MHz, CD_3OD, δ) 14.93, 17.99 (d, $^3J_{PC}$ = 5.75 Hz), 20.56 (d, $^1J_{PC}$ = 43.36 Hz), 27.00 (s, 6Me), 35,71 (d, $^1J_{PC}$ = 38.94 Hz), 58.63 (d, $^1J_{PC}$ = 83.18 Hz, $C_{Cp,}$*ipso*), 70.93 (s, $C_{Cp'}$), 73.03 (d, $^3J_{CP}$ = 9.05 Hz), 73.15 (d, $^2J_{CP}$ = 8.75 Hz). Anal. calc. (%): C, 55.65; H, 7.56; Br, 17.63; Fe, 12.32; P, 6.83. Found: C, 54.59; H, 7.42; Br, 18.96; Fe, 12.25; P, 6.78
Hexyl(di-*tert*-butyl)ferrocenylphosphonium bromide **(4c)**.
Dark orange solid 0.449 g (71 %), m.p. 67-68 °C%). ^{31}P NMR (162 MHz, C_6D_6, δ) 47.85; ^{1}H NMR (400 MHz, DMSO-d_6, δ) 0.93 (t, $^3J_{HH}$ = 6.91 Hz, 3H), 1.27 (m, 4H), 1.37 (d, $^3J_{PH}$ = 15.08 Hz, 18H), 1.59 (m, 2H), 1.99 (m, 2H), 2.47 (m, 2H), 4.42 (s, 5H), 4.80 (s, 2H), 4.92 (s, 2H); ^{13}C{1H} NMR (100 MHz, C_6D_6, δ) 14.08, 20.12 (d, $^1J_{PC}$ = 54.25 Hz), 22.64, 24.79, 28.45(s, 6Me), 31.64 (d, $^2J_{PC}$ = 15.85 Hz), 31.90, 36.25 (d, $^1J_{PC}$ = 36.16 Hz), 50.86 (d, $^1J_{PC}$ = 97.68 Hz, $C_{Cp,}$*ipso*), 71.34 (s, $C_{Cp'}$), 73.19 (d, $^2J_{CP}$ = 7.53 Hz), 73.37 (d, $^3J_{CP}$ = 8.69 Hz). ESI-MS (m/z) 415.3 $[M]^+$. Anal. calc. (%): C, 58.20; H, 8.14; Br, 16.13; Fe, 11.28; P, 6.25. Found: C, 57.74; H, 8.05; Br, 16.62; Fe, 11.27; P, 6.32.
Decyl(di-*tert*-butyl)ferrocenylphosphonium bromide **(4d)**.
Brown glass 0.796 g (69 %), m.p. 58-70 °C. ^{31}P NMR (162 MHz, C_6D_6, δ) 47.45; ^{1}H NMR (400 MHz, C_6D_6, δ) 0.84 (t, $^3J_{HH}$ = 7.11 Hz, 3H), 1.24 (m, 12H), 1.38 (d, $^3J_{PH}$ = 15.08 Hz, 18H), 1.61 (m, $^3J_{HH}$ = 7.47 Hz, 2H), 2.00 (m, 2H), 2.43 (m, 2H), 4.32 (s, 5H), 4.49 (s, 2H), 4.80 (s, 2H); ^{13}C{1H} NMR (100 MHz, C_6D_6, δ) 13.93, 19.26 (d, $^1J_{PC}$ = 42.07 Hz), 22.55, 24.48 (d, $^3J_{PC}$ = 5.80 Hz), 28.03 (s, 6Me), 29.18, 29.30, 29.36, 29.44, 30,79 31.65 (d, $^2J_{PC}$ = 14.00 Hz), 31.76, 36.11 (d, $^1J_{PC}$ = 38.29 Hz), 58.89 (d, $^1J_{PC}$ = 80.18 Hz, $C_{Cp,}$*ipso*), 70.86 (s, $C_{Cp'}$), 72.89 (d, $^3J_{CP}$ = 8.78 Hz), 73.45 (d, $^2J_{CP}$ = 8.66 Hz). ESI-MS (m/z) 471.4 $[M]^+$. Anal. calc. (%): C, 60.99; H, 8.77; Br, 14.49; Fe, 10.13; P, 5.62. Found: C, 61.97; H, 9.36; Br, 13.74; Fe, 9.60; P, 5.33.
Tetradecyl(di-*tert*-butyl)ferrocenylphosphonium bromide **(4e)**.
Brown solid, yield 0.418 g (59%), m.p. 61-71 °C. ^{31}P NMR (162 MHz C_6D_6, δ) 47.76; ^{1}H NMR (400 MHz, DMSO-d_6, δ) 0.85 (t, $^3J_{HH}$ = 7.44, 3H), 1.25 (m, 20H), 1.37 (d, $^3J_{PH}$ = 14.94 Hz, 18H), 1.58 (m, $^3J_{HH}$ = 7.47 Hz), 1.99 (m, 2H), 2.58 (m, 2H), 4.42 (s, 5H), 4.80 (s, 2H), 4.92 (s, 2H); ^{13}C{1H} NMR (100 MHz, C_6D_6, δ) 14.09, 19.81 (m), 22.64, 24.80, 28.46, 29.32, 29.47, 29.64, 31.65 (d, $^2J_{PC}$ = 15.98 Hz), 31.90, 36.29 (d, $^1J_{PC}$ = 36.42), 59,97 (d, $^1J_{PC}$ = 82.12 Hz), 71.34, 73.33. ESI-MS (m/z) 527.5 [M]. Anal. calc. (%): C, 63.27; H, 9.29; Br, 13.15; Fe, 9.19; P, 5.10. Found: C, 63.27; H, 9.29; Br, 13.15; Fe, 9.19; P, 5.10
General procedure for the synthesis of methyl-, propyl-, hexyl-, decyl-, tetradecyl-(di-*tert*-butyl)ferrocenylphosphonium tetrafluoroborates (**5a-e**). Salts (**4a-e**) were dissolved in 10 ml of ethyl alcohol, a twofold excess of sodium tetrafluoroborate solution in ethyl alcohol was added to them. The reaction mixture was stirred for

12 hours. The solvent was evaporated in vacuo, and the salt was dissolved in 20 ml of methylene chloride and washed with distilled water. The organic phase was separated and dried over magnesium sulfate. The solvent is evaporated, and the salt is dried under vacuum at 50 ° C for 8 hours.

Methyl(di-*tert*-butyl)ferrocenylphosphonium tetrafluoroborate **(5a).**

Orange solid, yield 0.312 g (86 %), m.p. 69-80 °C. ^{31}P NMR (162 MHz, $CDCl_3$, δ) 49.54; ^{1}H NMR (400 MHz, $CDCl_3$, δ) 1.47 (d, $^{3}J_{PH}$ = 15.48 Hz, 18H), 2.18 (d, $^{2}J_{PH}$ =10.44 Hz, 3H), 4.43 (s, 5H), 4.63 (s, 2H), 4.82 (s, 2H). ESI-MS (m/z) 345.2 $[M]^+$, 87.2 $[BF_4]^-$. Anal. calc. (%): C, 52.82; H, 7.00; B, 2.50; F, 17.59; Fe, 12.93; P, 7.17. Found: C - 54.23; H - 7.65; B - 2.39; Fe - 12.21; P - 6.58.

Propyl(di-*tert*-butyl)ferrocenylphosphonium tetrafluoroborate **(5b)**.

Orange solid 0.472 g (82 %), m.p. 57-67 °C.^{31}P NMR (162 MHz, $CDCl_3$, δ) 47.53; ^{1}H NMR (400 MHz, $CDCl_3$, δ) 1.34 (t, $^{4}J_{PH}$ = 2.17 Hz, $^{3}J_{HH}$ = 7.18 Hz, 3H), 1.47 (dt, $^{3}J_{PH}$ = 15.27 Hz, 18H), 2.13 (m, 2H), 2.51 (m, 2H), 4.40 (s, 5H), 4.62 (s, 2H), 4.86 (s, 2H). ESI-MS (m/z) 373.24 $[M-HBF_4]^+$, 87.2 $[BF_4]^-$. Anal. calc. (%) C, 54.82; H, 7.45; B, 2.35; F, 16.52; Fe, 12.14; P, 6.73. Found: C, 53.97; H, 6.58; B, 2.16; Fe, 11.16; P, 6.19.

Hexyl(di-*tert*-butyl)ferrocenylphosphonium tetrafluoroborate **(5c)**.

Brown solid, yield 0.472 g (81 %), m.p. 51-52 °C. ^{31}P NMR (162 MHz, $CDCl_3$, δ) 48.11 (s); ^{1}H NMR (400 MHz, $CDCl_3$, δ) 0.97 (t, $^{3}J_{HH}$ = 6.02 Hz, 3H), 1.28 (m, 4H), 1.48 (d, $^{3}J_{PH}$ = 15.28 Hz, 18H), 1.70 (m, 2H), 2.07 (m, 2H), 2.52 (m, 2H), 4.40 (s, 5H), 4.62 (s, 2H), 4.87 (s, 2H). ESI-MS (m/z) 415.3 $[M]^+$, 87.2 $[BF_4]^-$ Anal. calc. (%): C, 57.40; H, 8.03; B, 2.15; F, 15.13; Fe, 11.12; P, 6.17. Found: C, 56.21; H, 7.62; B, 2.15; Fe, 10.49; P, 6.16.

Decyl(di-*tert*-butyl)ferrocenylphosphonium tetrafluoroborate **(5d)**.

Dark orange solid, yield 0.538 g (89 %), m.p. 34-38 °C. ^{31}P NMR (162 MHz, $CDCl_3$, δ) 48.33; ^{1}H NMR (400 MHz, $CDCl_3$, δ) 0.90 (t, $^{3}J_{HH}$ = 7.01 Hz, 3H), 1.28 (m, 12H), 1.49 (d, $^{3}J_{PH}$ = 14.82, 18H), 1.71 (m, 2H), 2.07 (m, 2H), 2.56 (m, 2H), 4.41 (s, 5H), 4.64 (s, 2H), 4.87 (s, 2H). ESI-MS (m/z) 471.4 $[M]^+$, 87.2 $[BF_4]^-$. Anal. Calc. (%): C, 60.24; H, 8.67; B, 1.94; F, 13.61; Fe, 10.00; P, 5.55. Found: C, 61.24; H, 9.25; B, 1.84; Fe, 9.49; P, 5.25.

Tetradecyl(di-*tert*-butyl)ferrocenylphosphonium tetrafluoroborate **(5e)**.

Brown solid, yield 0.493 g (81 %), m.p. 57-60 °C. ^{31}P NMR (162 MHz, $CDCl_3$, δ) 48.55; ^{1}H NMR (400 MHz, $CDCl_3$, δ) 0.90 (t, $^{3}J_{HH}$ = 6.74 Hz, 3H), 1.30 (m, 20H), 1.48 (d, $^{3}J_{PH}$ = 15.15 Hz, 18H), 1.67 (m, 2H), 2.15 (m, 2H), 2.57 (m, 2H), 4.45 (s, 5H), 4.78 (s, 2H), 4.95 (s, 2H). ESI-MS 527,5 $[M-]^+$, 87.2 $[BF_4]^-$. Anal. Calc. (%):C, 62.55; H, 9.19; B, 1.76; F, 12.37; Fe, 9.09; P, 5.04. Found: C, 63.35; H, 9.70; B, 1.68; Fe, 8.67; P, 4.81.

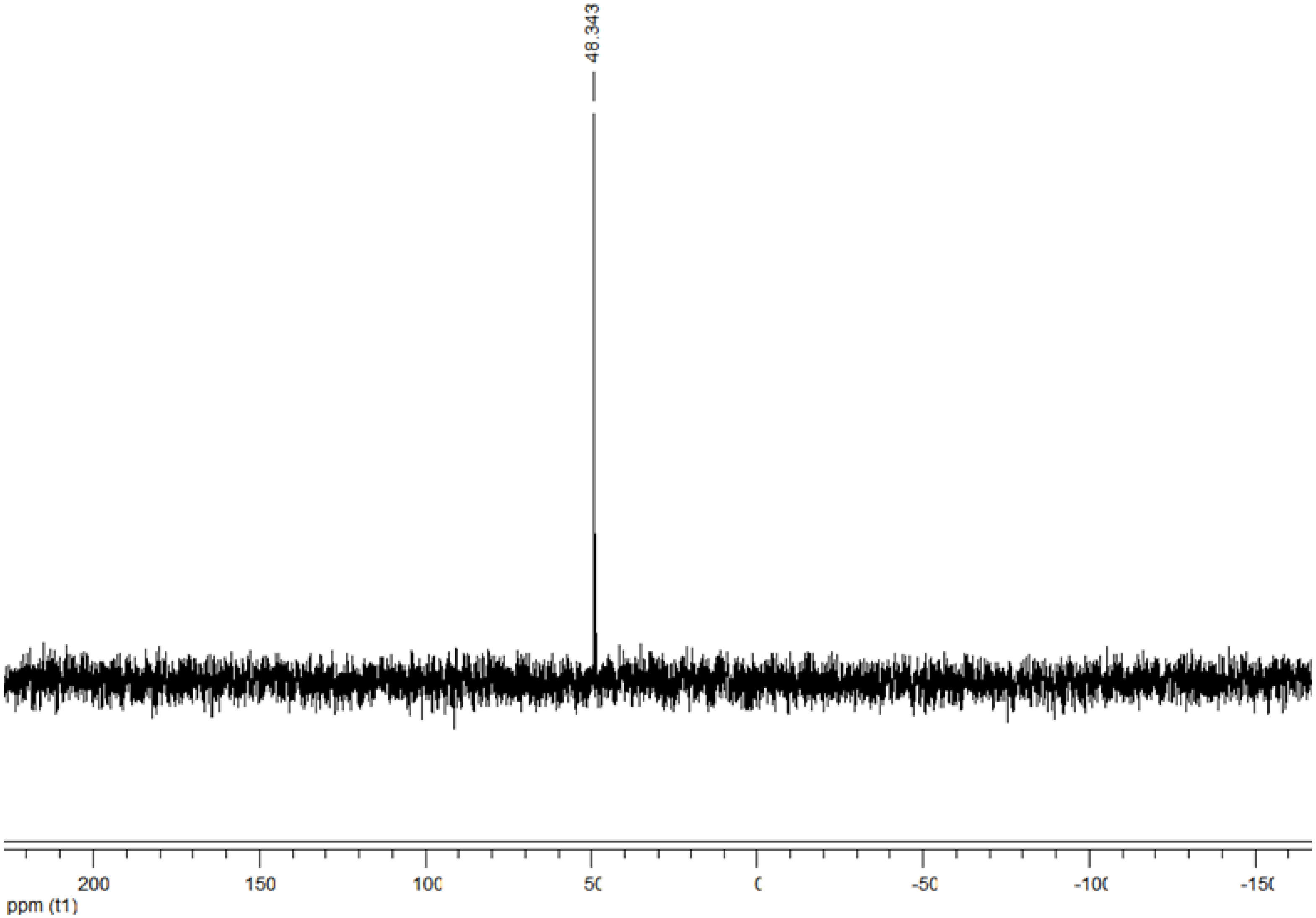

Fig.1.^{31}P NMR spectra of the methyl(di-*tert*-butyl)ferrocenylphosphonium iodide **(4a)**

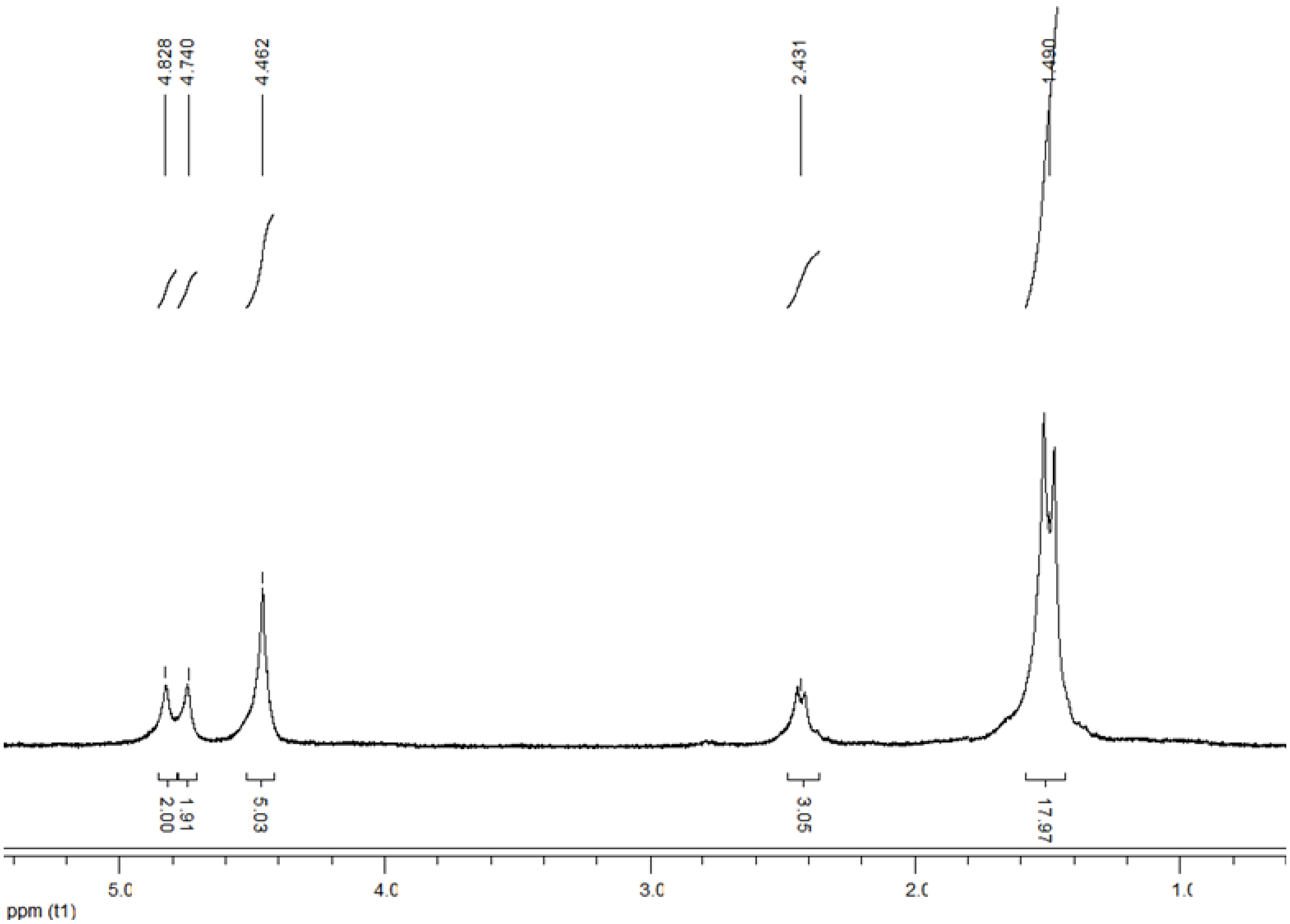

Fig.2. ^{1}H NMR spectra of the methyl(di-*tert*-butyl)ferrocenylphosphonium iodide **(4a)**

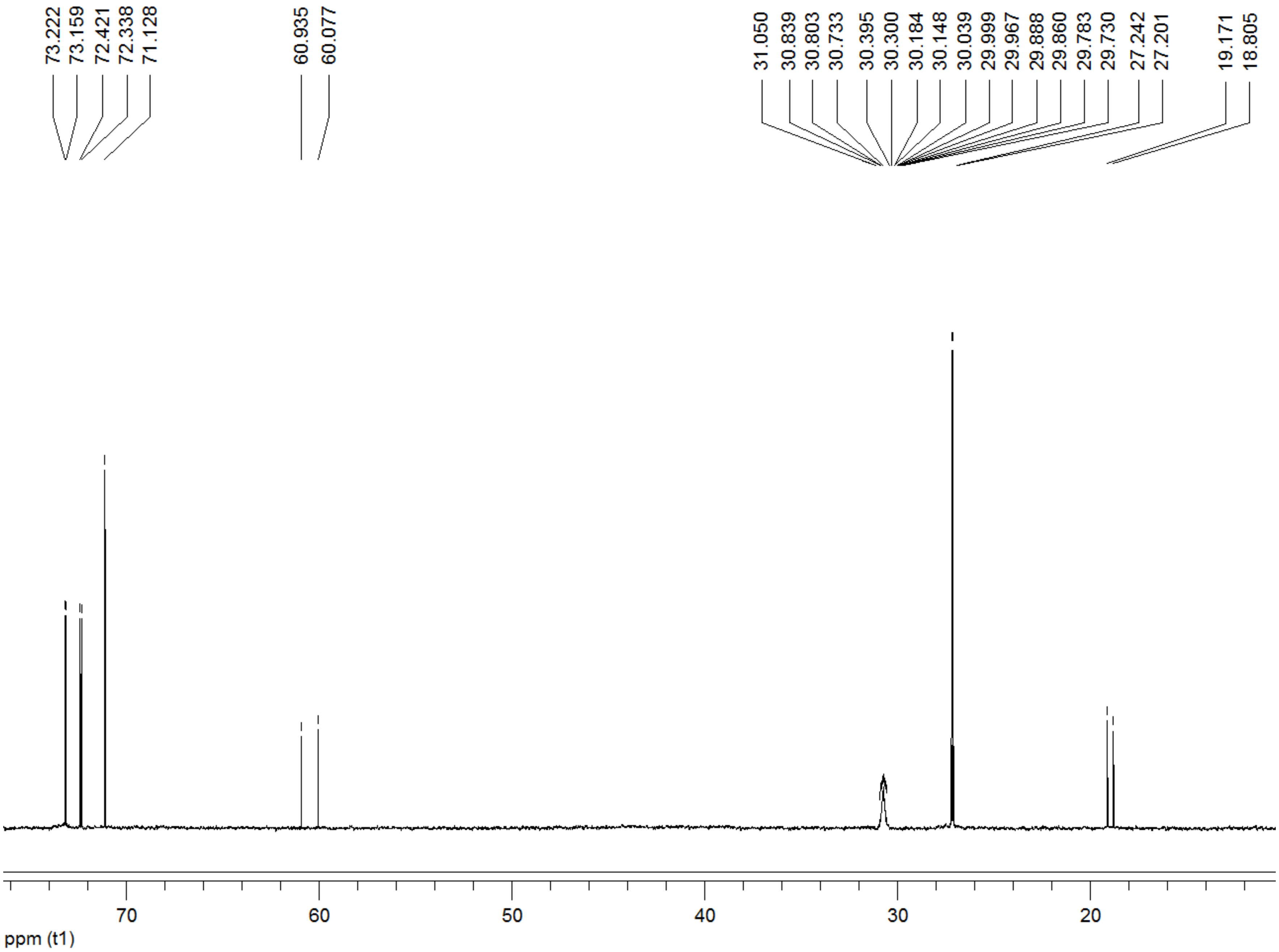

Fig.3. $^{13}C\{^1H\}$ NMR spectra of the methyl(di-*tert*-butyl)ferrocenylphosphonium iodide **(4a)**

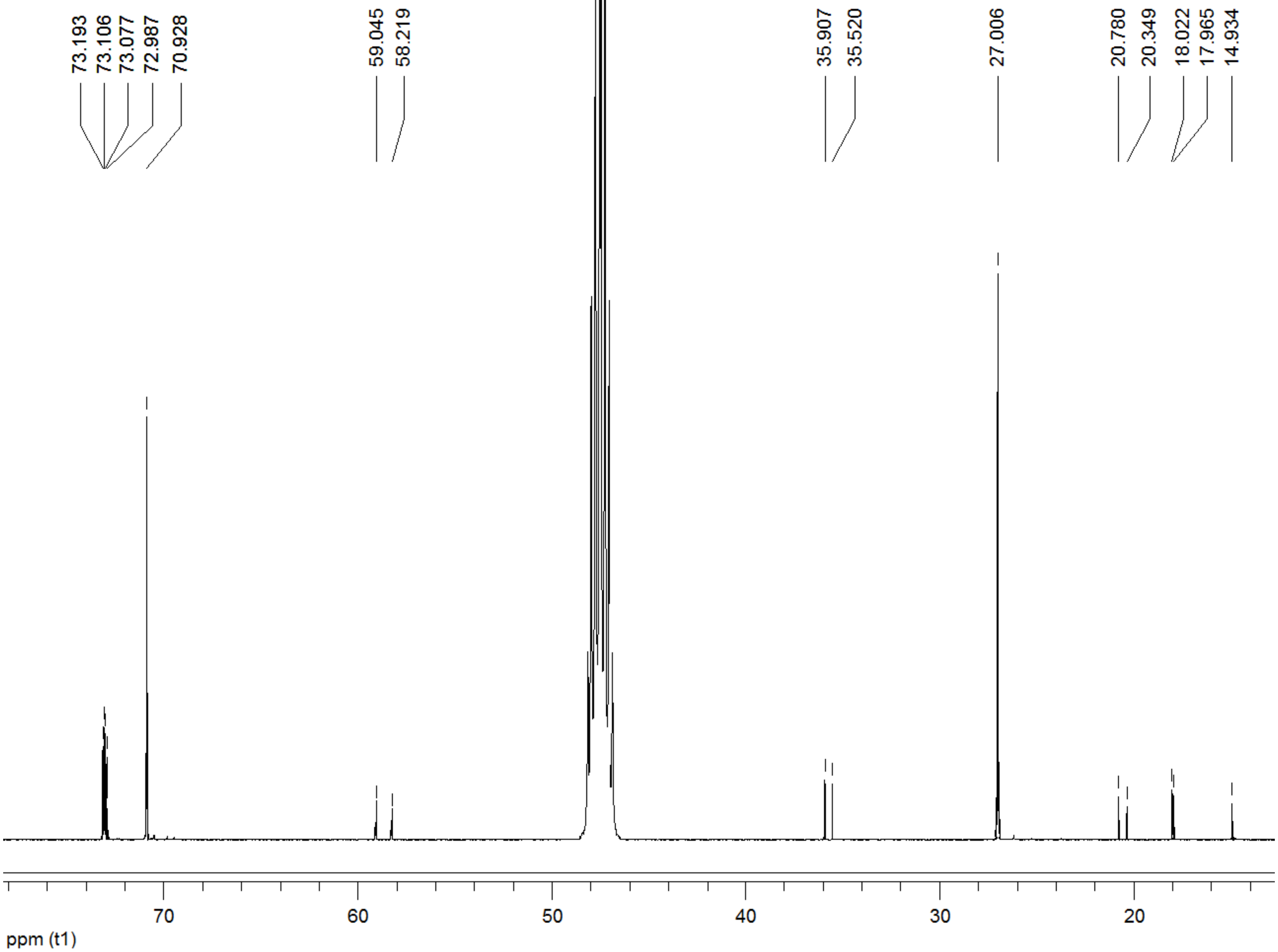

Fig.4. $^{13}C\{^{1}H\}$ NMR spectra of the propyl(di-*tert*-butyl)ferrocenylphosphonium bromide **(4a)**

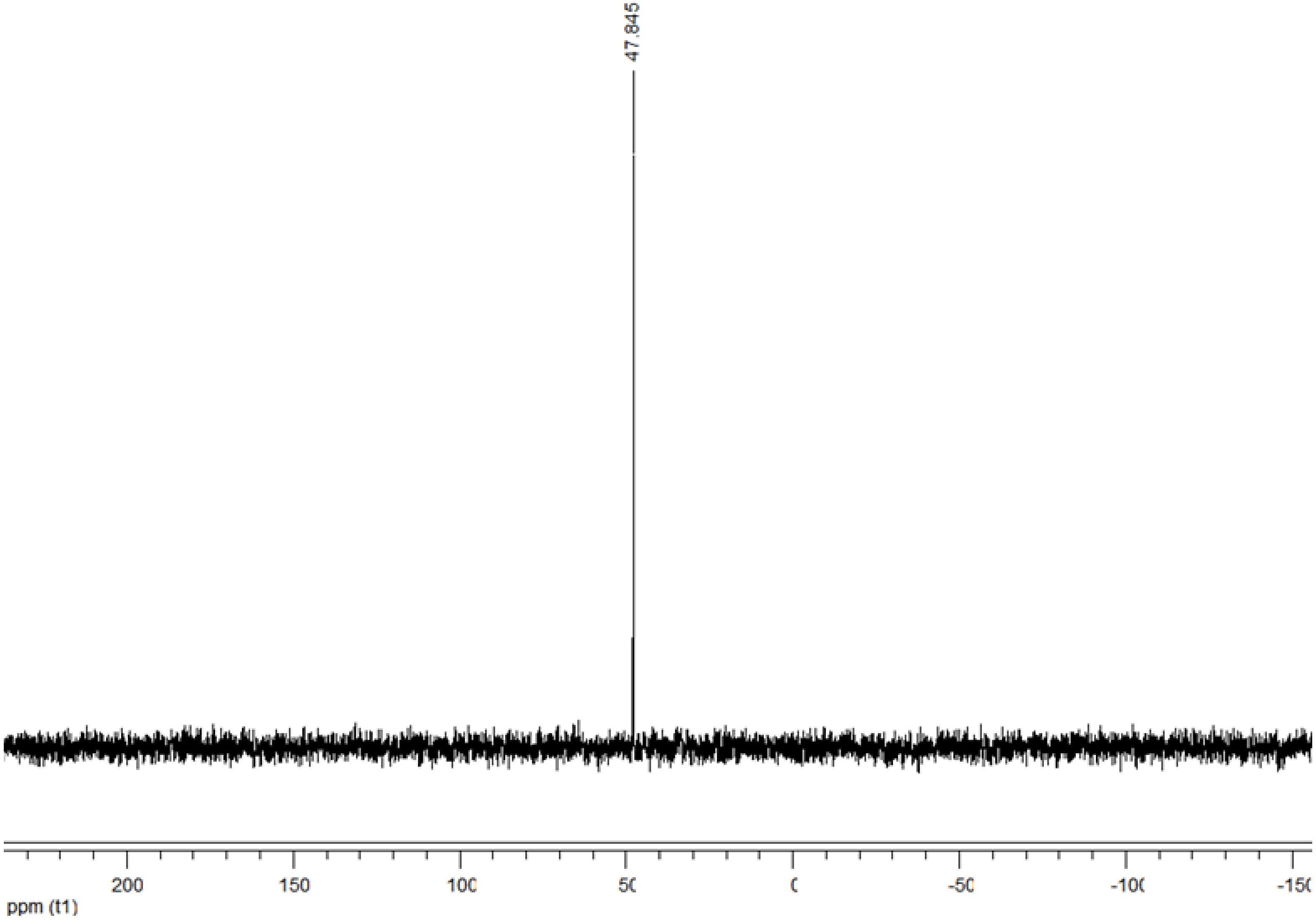

Fig.5. ^{31}P NMR spectra of the hexyl(di-*tert*-butyl)ferrocenylphosphonium bromide (**4c**)

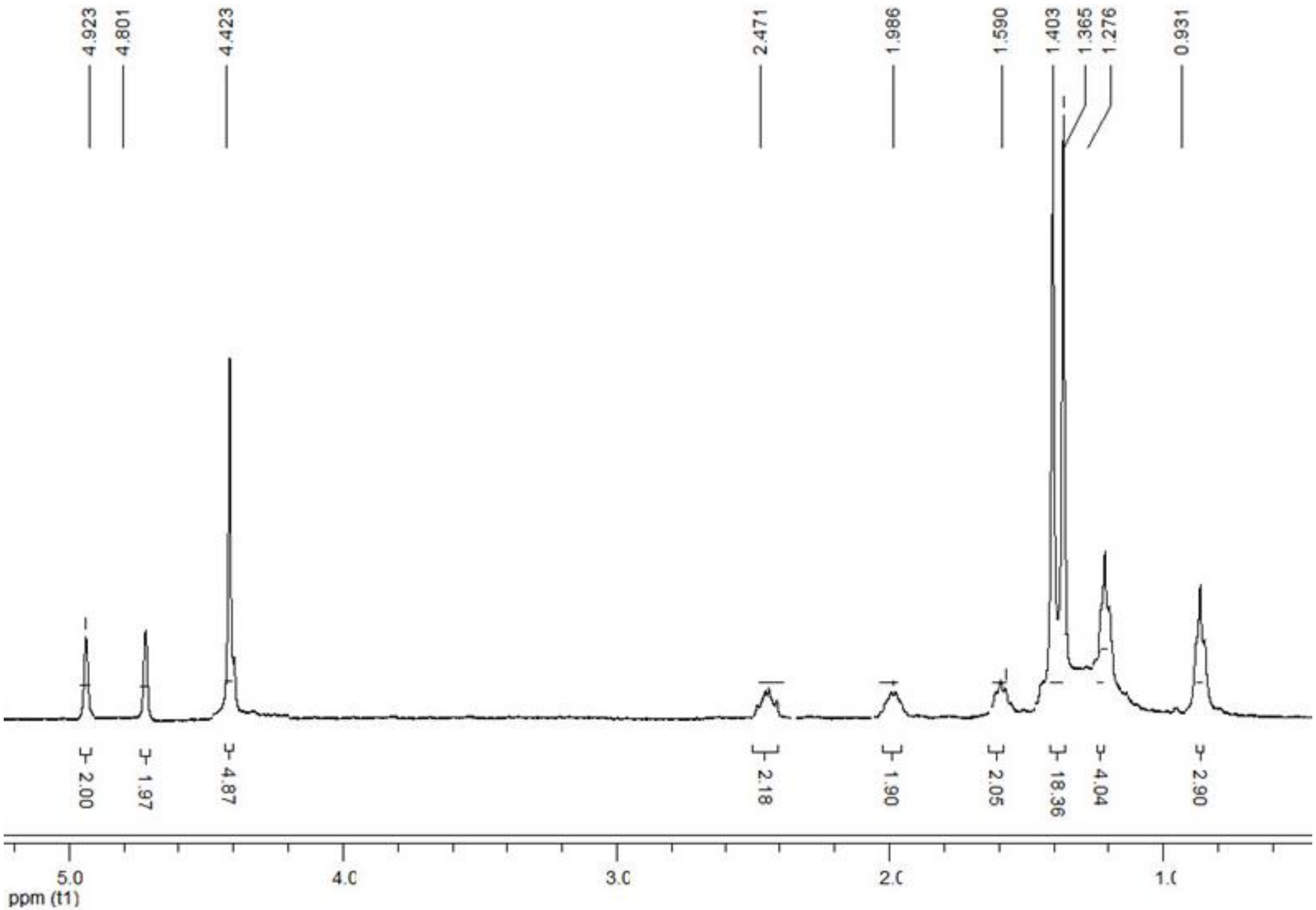

Fig.6.^{1}H NMR spectra of the hexyl(di-*tert*-butyl)ferrocenylphosphonium bromide (**4c**)

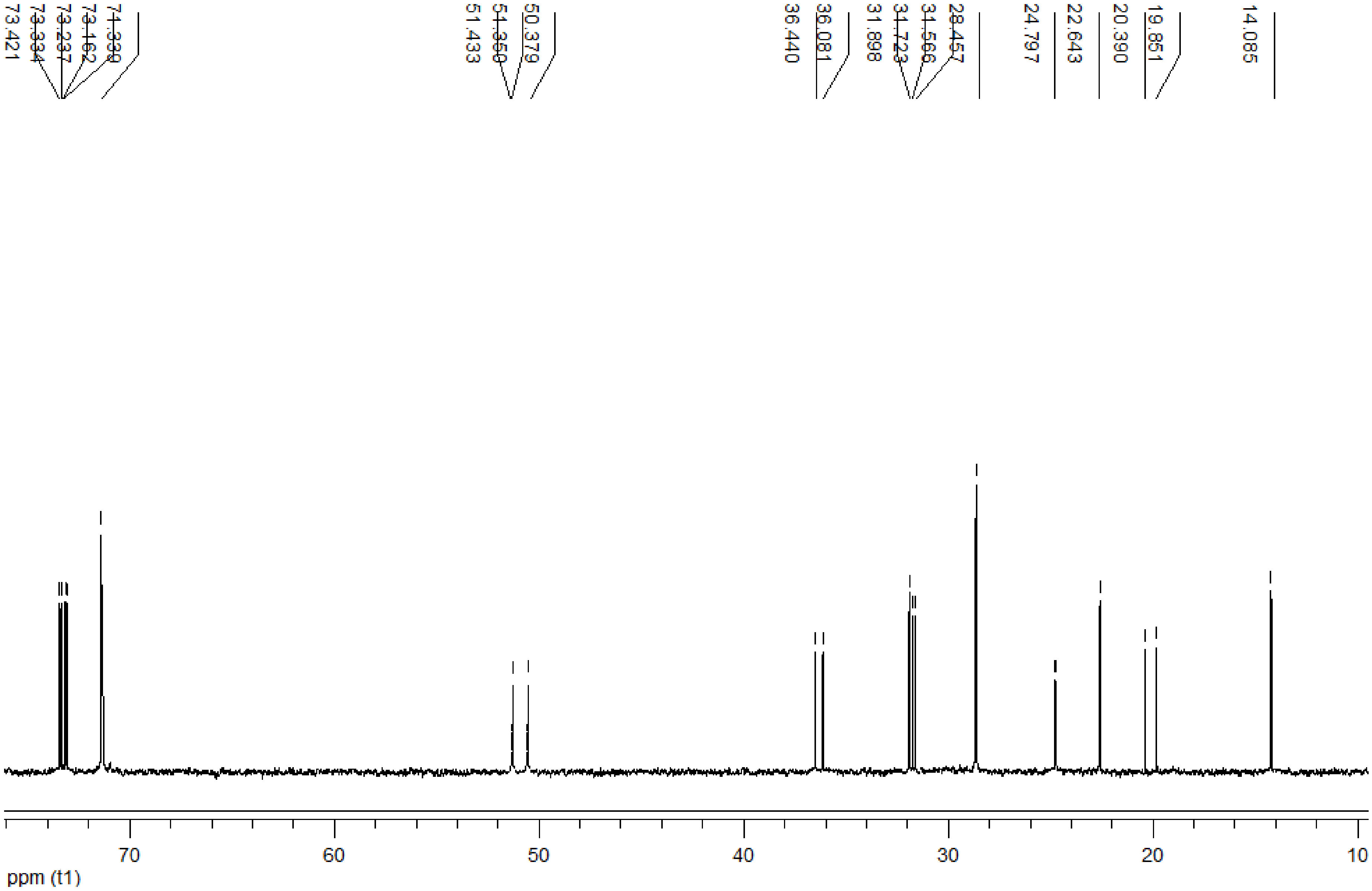

Fig.7. ^{13}C NMR spectra of the hexyl(di-*tert*-butyl)ferrocenylphosphonium bromide (**4c**)

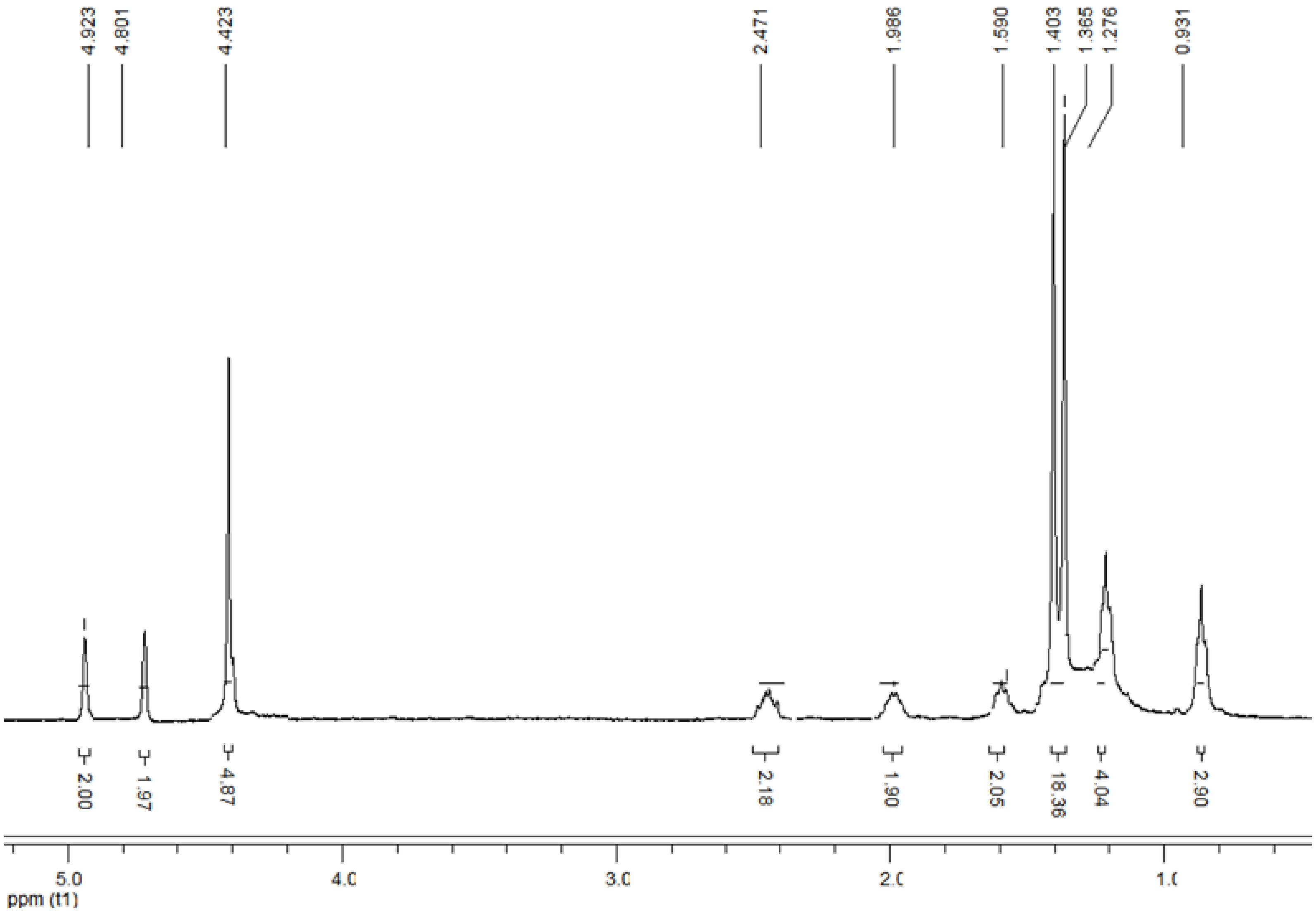

Fig.8. ^{1}H NMR spectra of the decyl(di-*tert*-butyl)ferrocenylphosphonium bromide (**4d)**

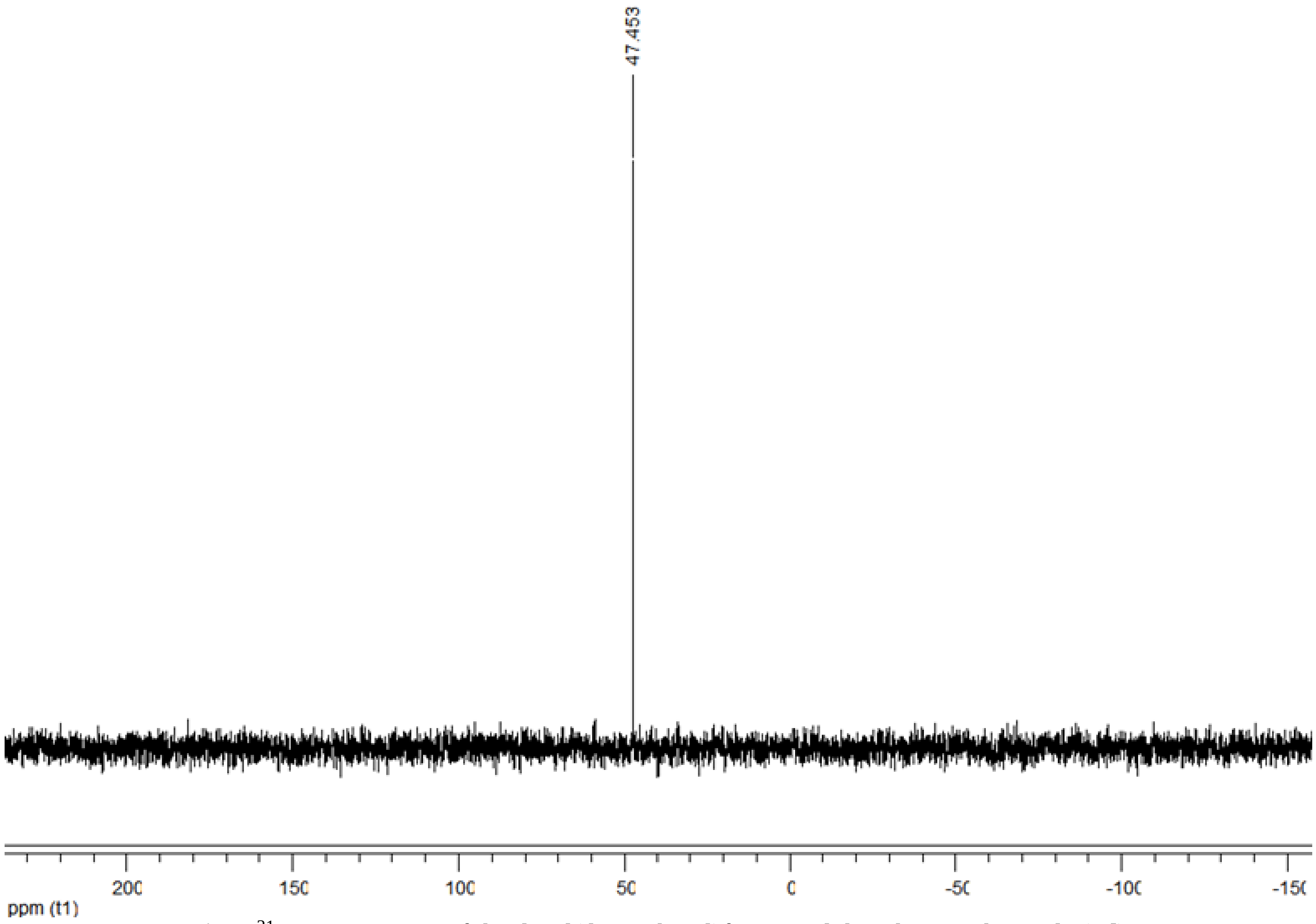

Fig.9. ^{31}P NMR spectra of the decyl(di-*tert*-butyl)ferrocenylphosphonium bromide (**4d)**

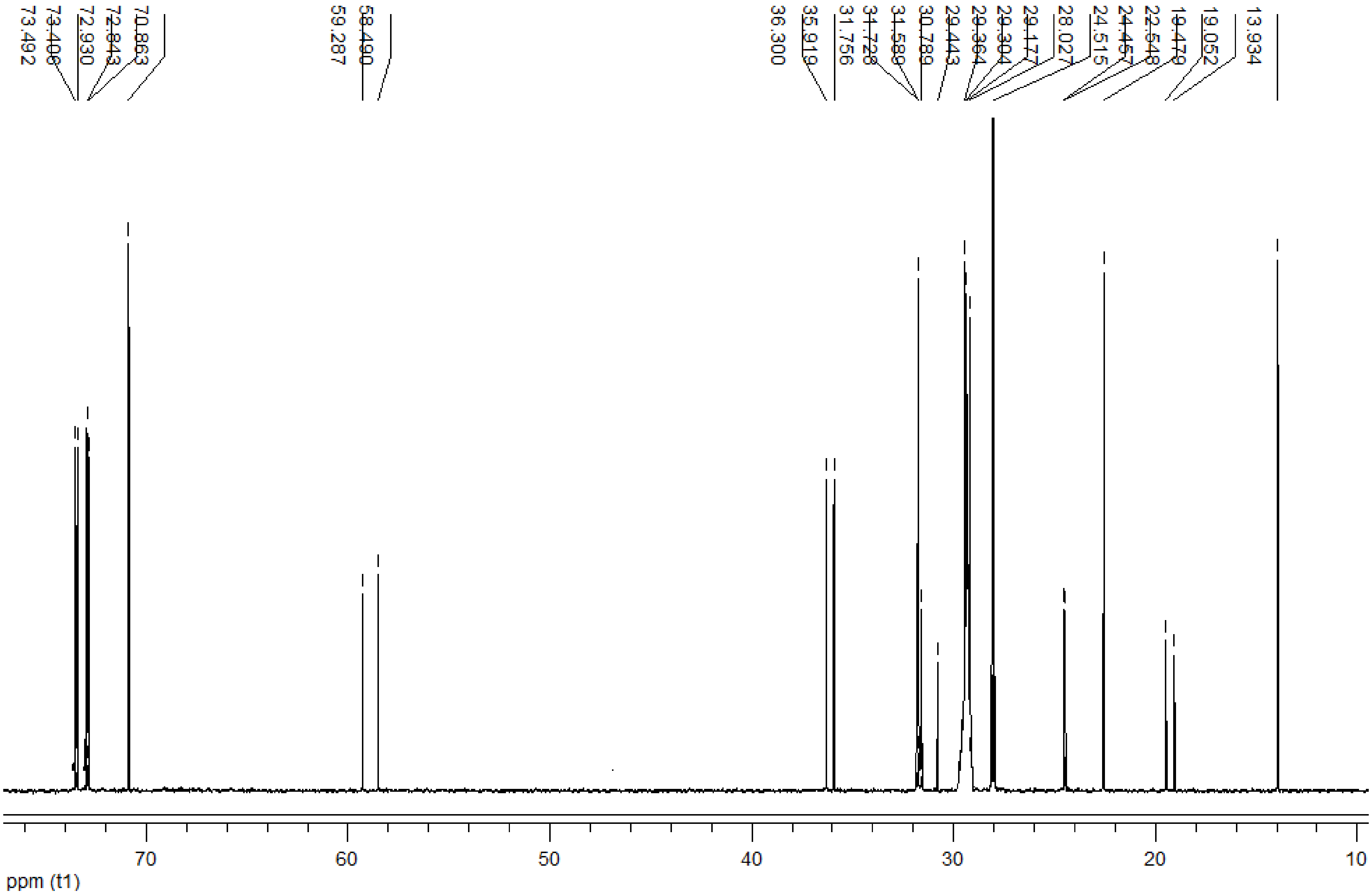

Fig.10.^{13}C NMR spectra of the decyl(di-*tert*-butyl)ferrocenylphosphonium bromide (**4d**)

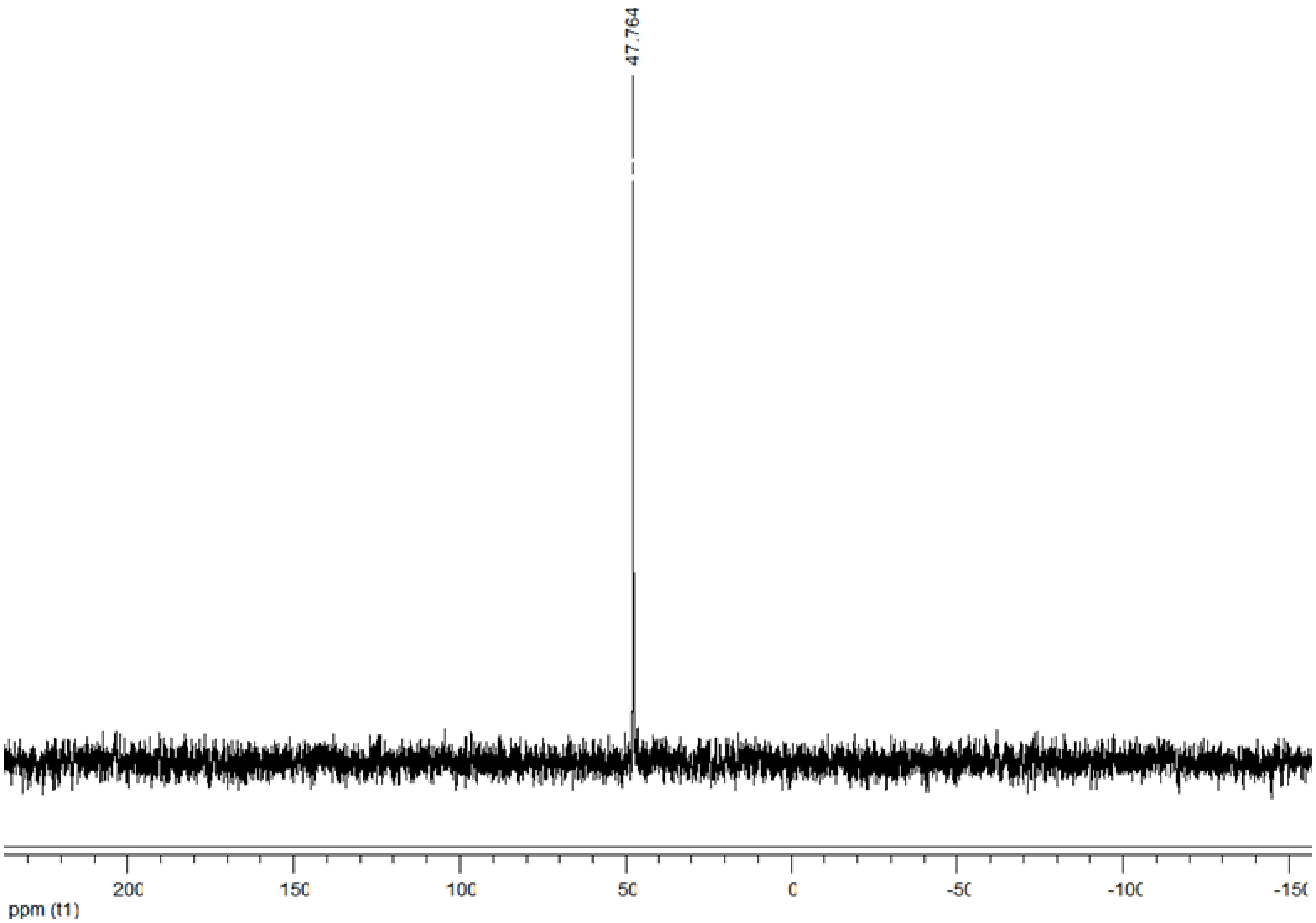

Fig.11.^{31}P NMR spectra of the tetradecyl(di-*tert*-butyl)ferrocenylphosphonium bromide (**4e**)

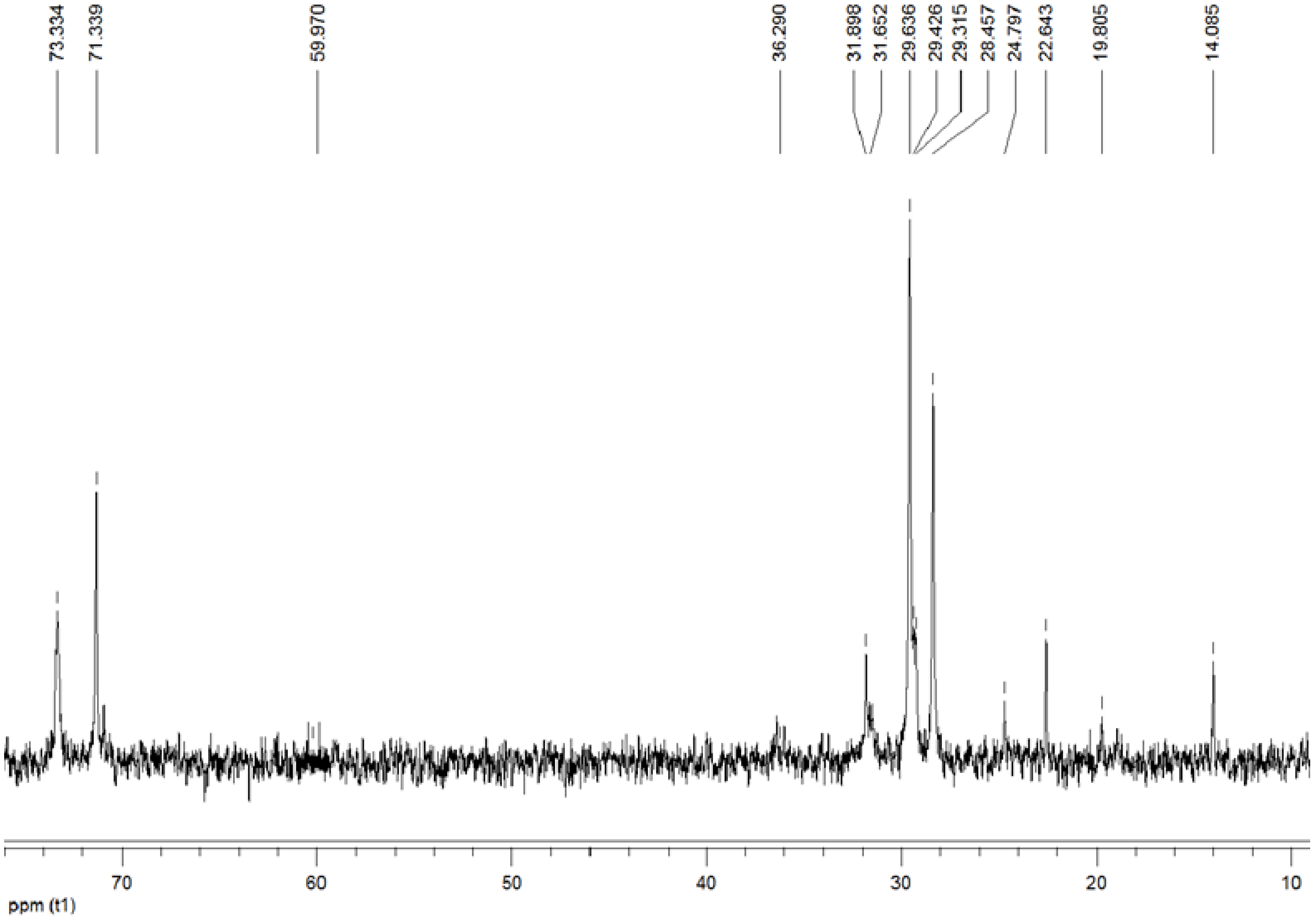

Fig.12.^{13}C NMR spectra of the tetradecyl(di-*tert*-butyl)ferrocenylphosphonium bromide (**4e**)

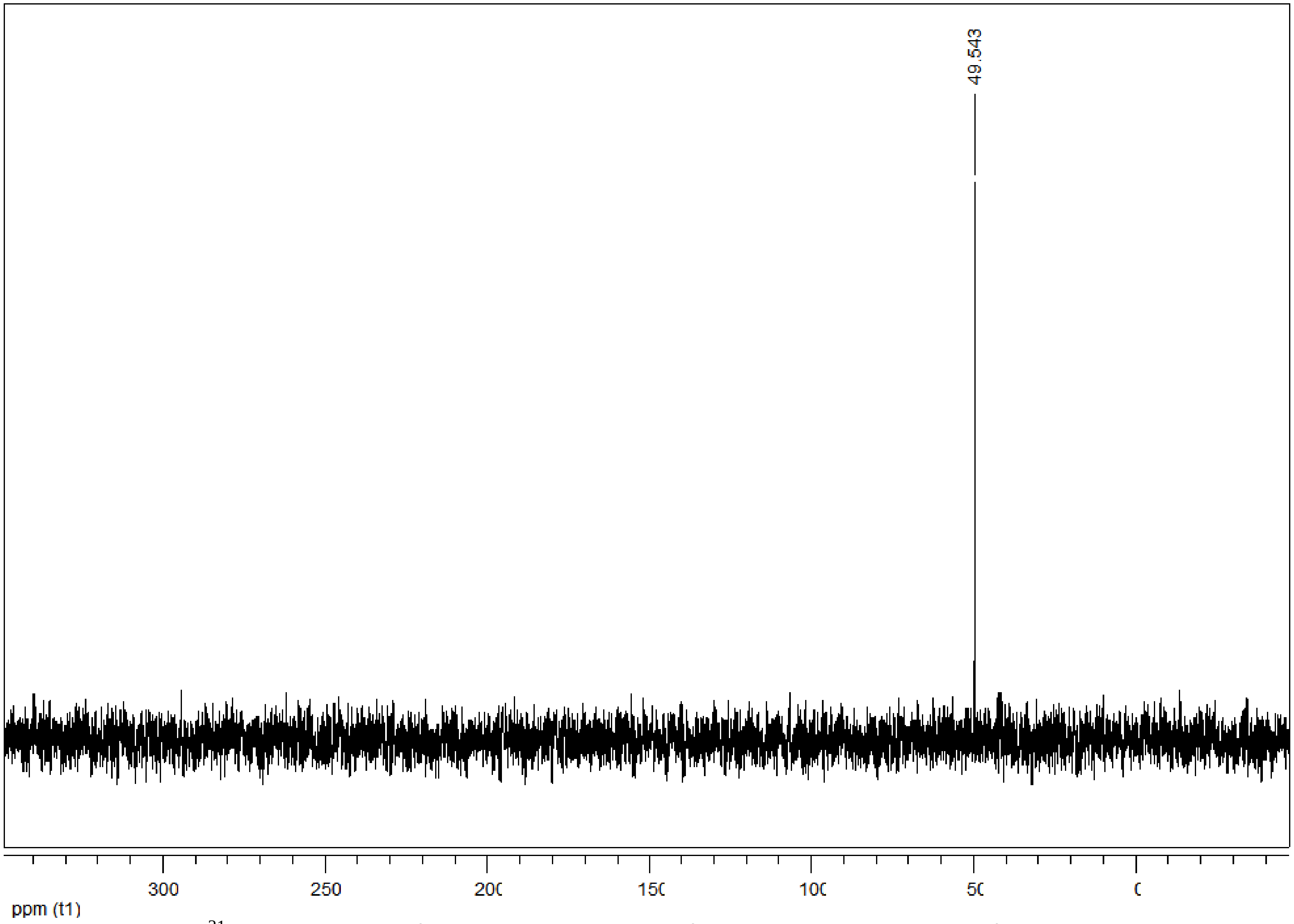

Fig.13. ^{31}P NMR spectra of the methyl(di-*tert*-butyl)ferrocenylphosphonium tetrafluoroborate (**5a**)

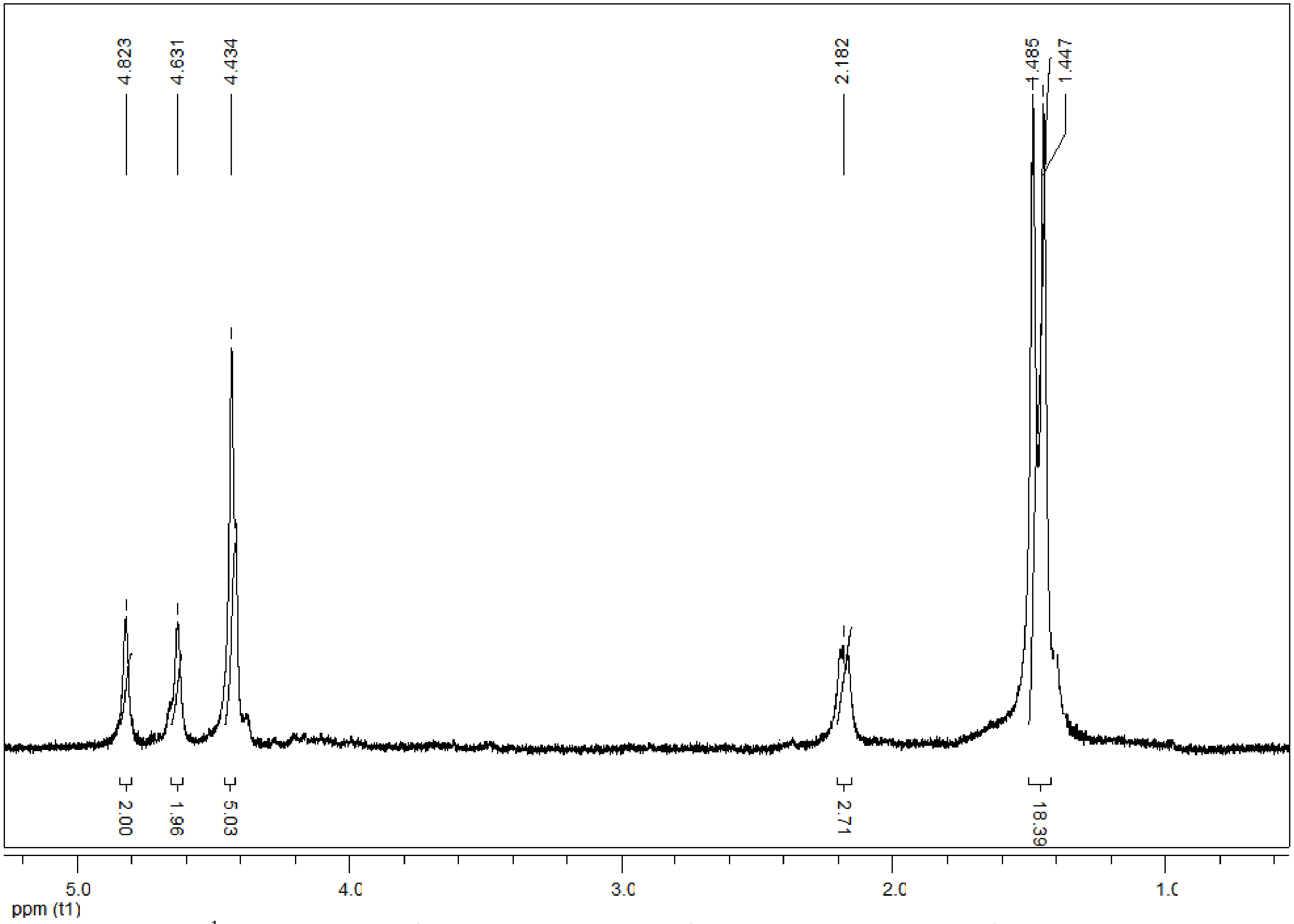

Fig.14. ^{1}H NMR spectra of the methyl(di-*tert*-butyl)ferrocenylphosphonium tetrafluoroborate (**5a**)

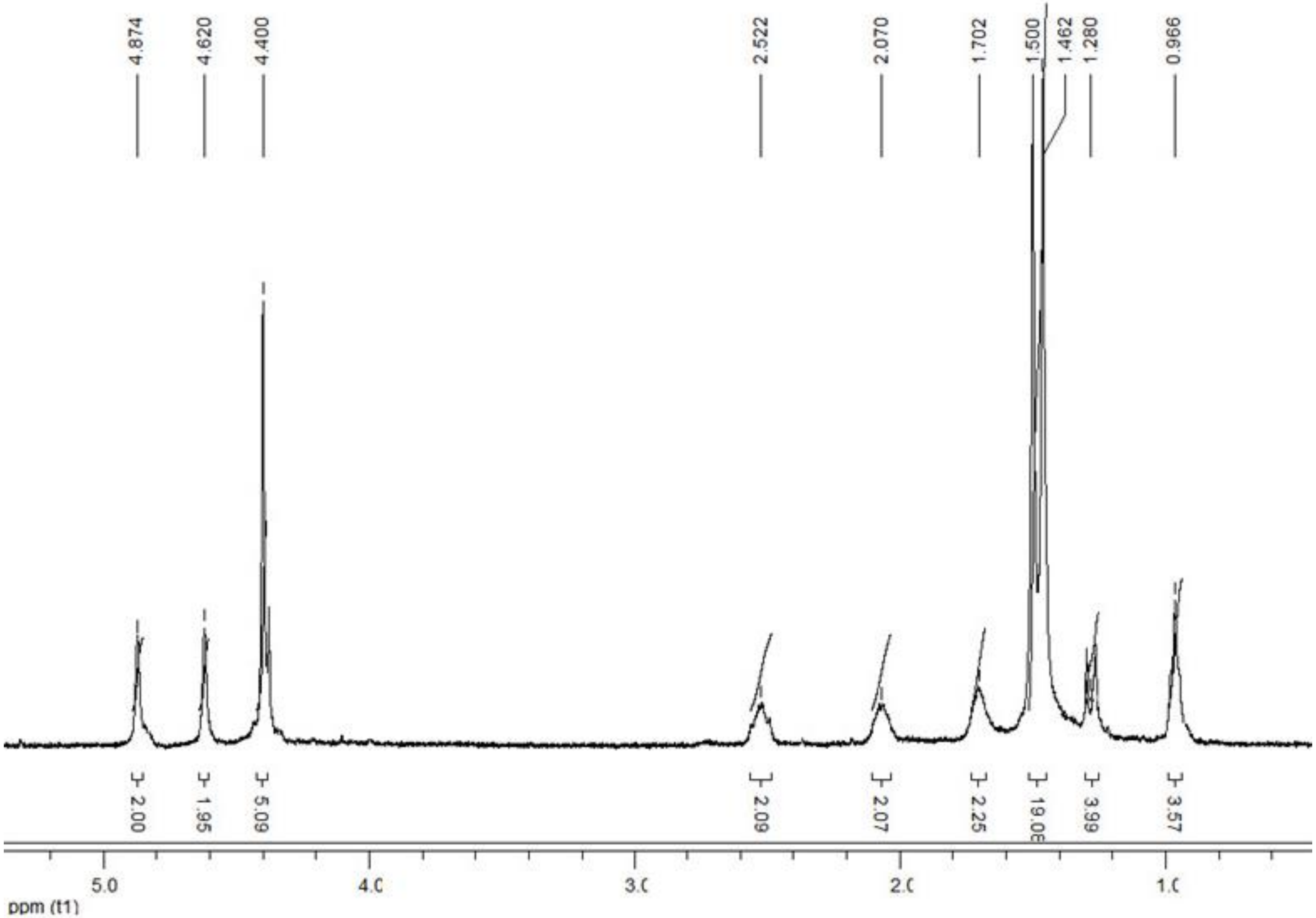

Fig.15.^{1}H NMR spectra of the hexyl(di-*tert*-butyl)ferrocenylphosphonium tetrafluoroborate (**5c)**

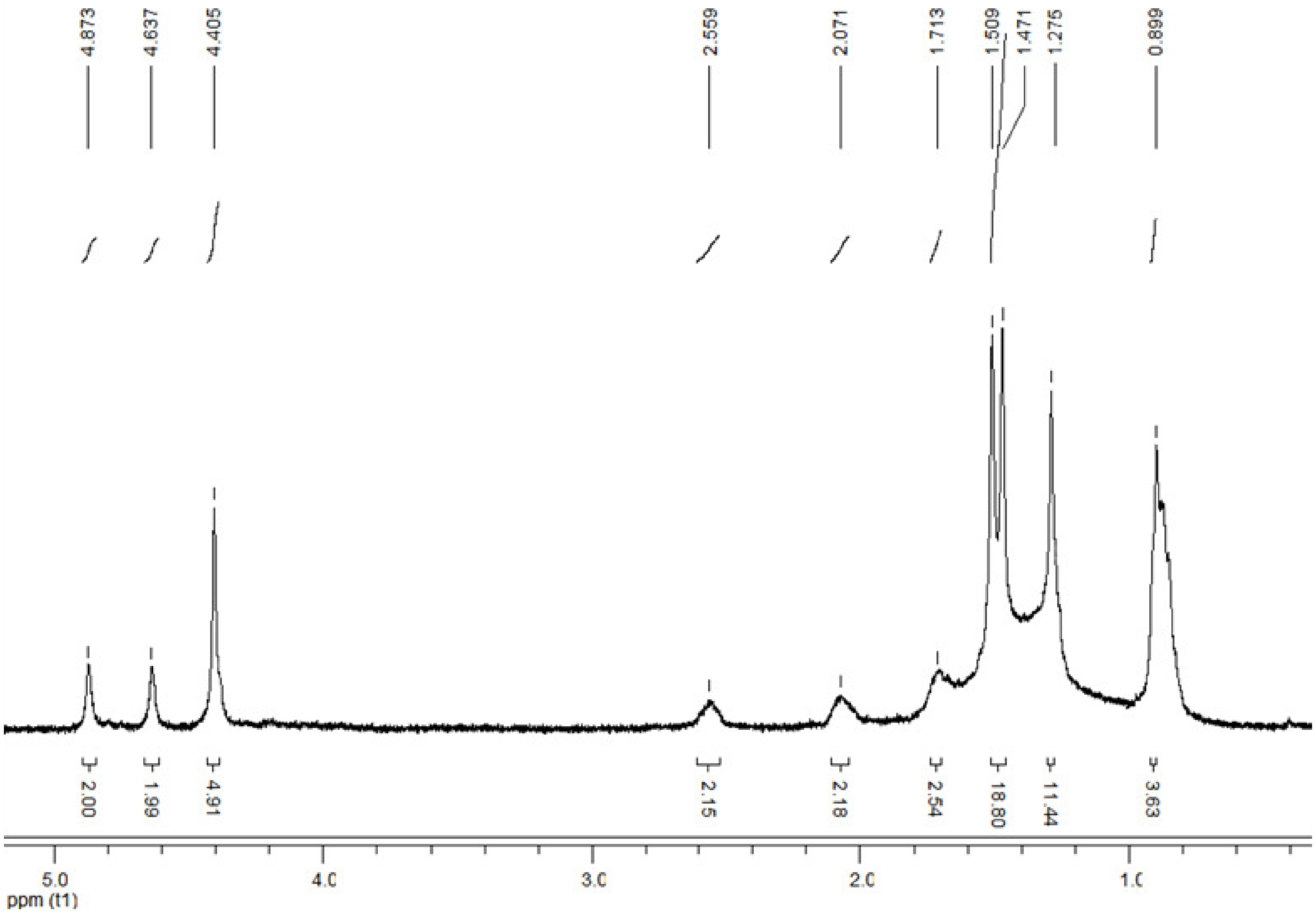

Fig.16.^{1}H NMR spectra of the decyl(di-*tert*-butyl)ferrocenylphosphonium tetrafluoroborate (**5d)**

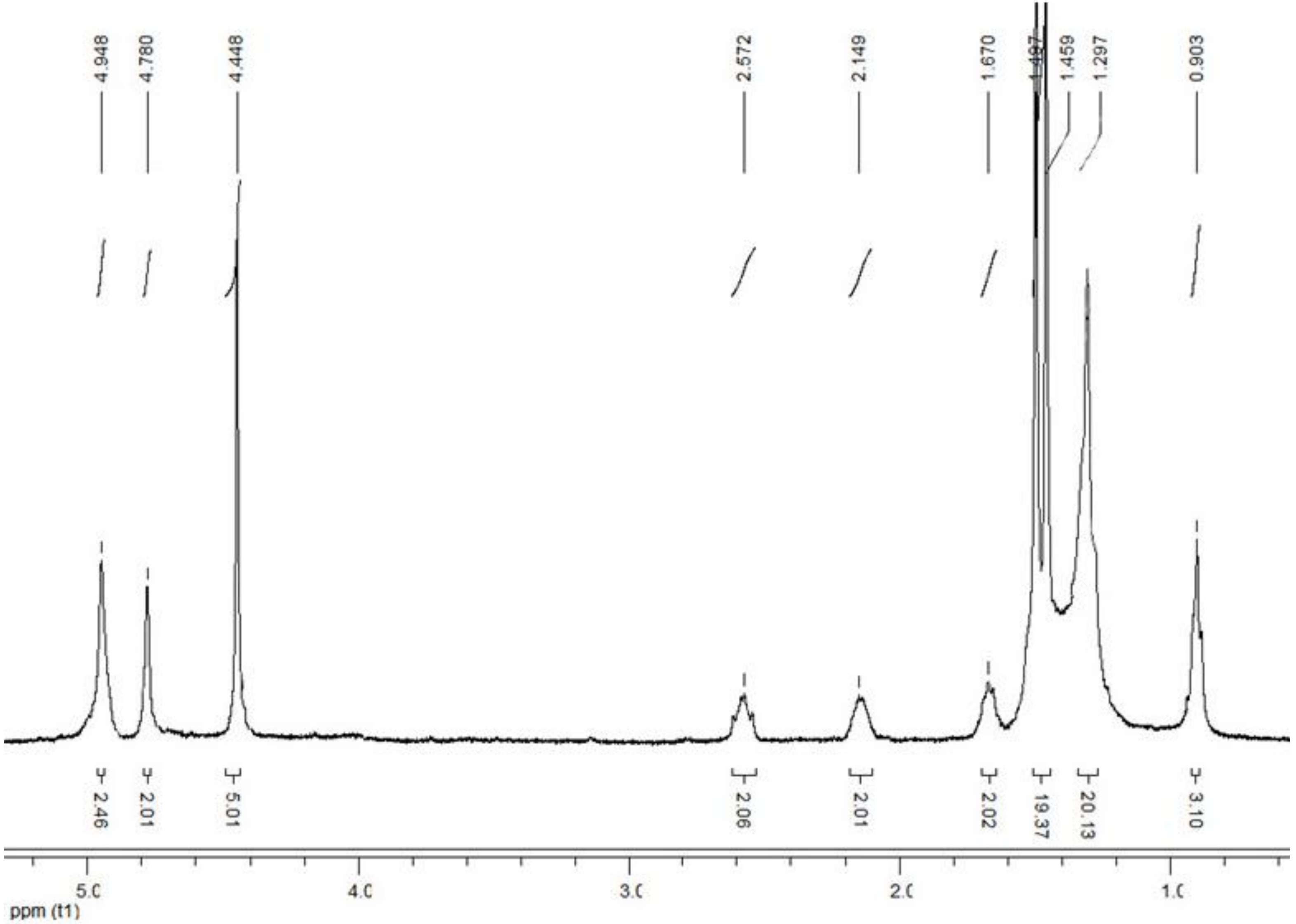

Fig.17. ^{1}H NMR spectra of the tetradecyl(di-*tert*-butyl)ferrocenylphosphonium tetrafluoroborate **5e**

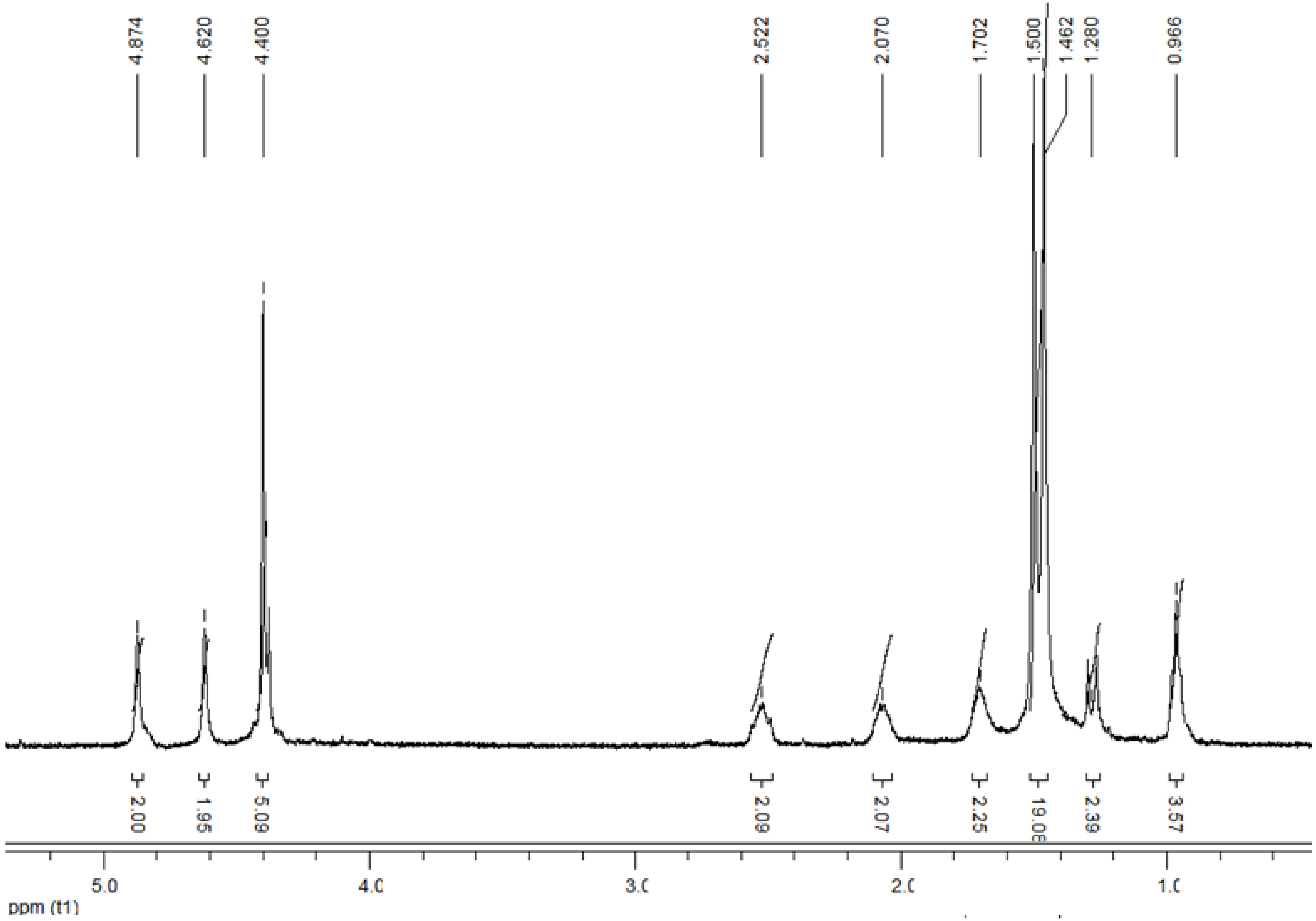

Fig.18.^{1}H NMR spectra of the hexyl(di-*tert*-butyl)ferrocenylphosphonium tetrafluoroborate (**5c**)

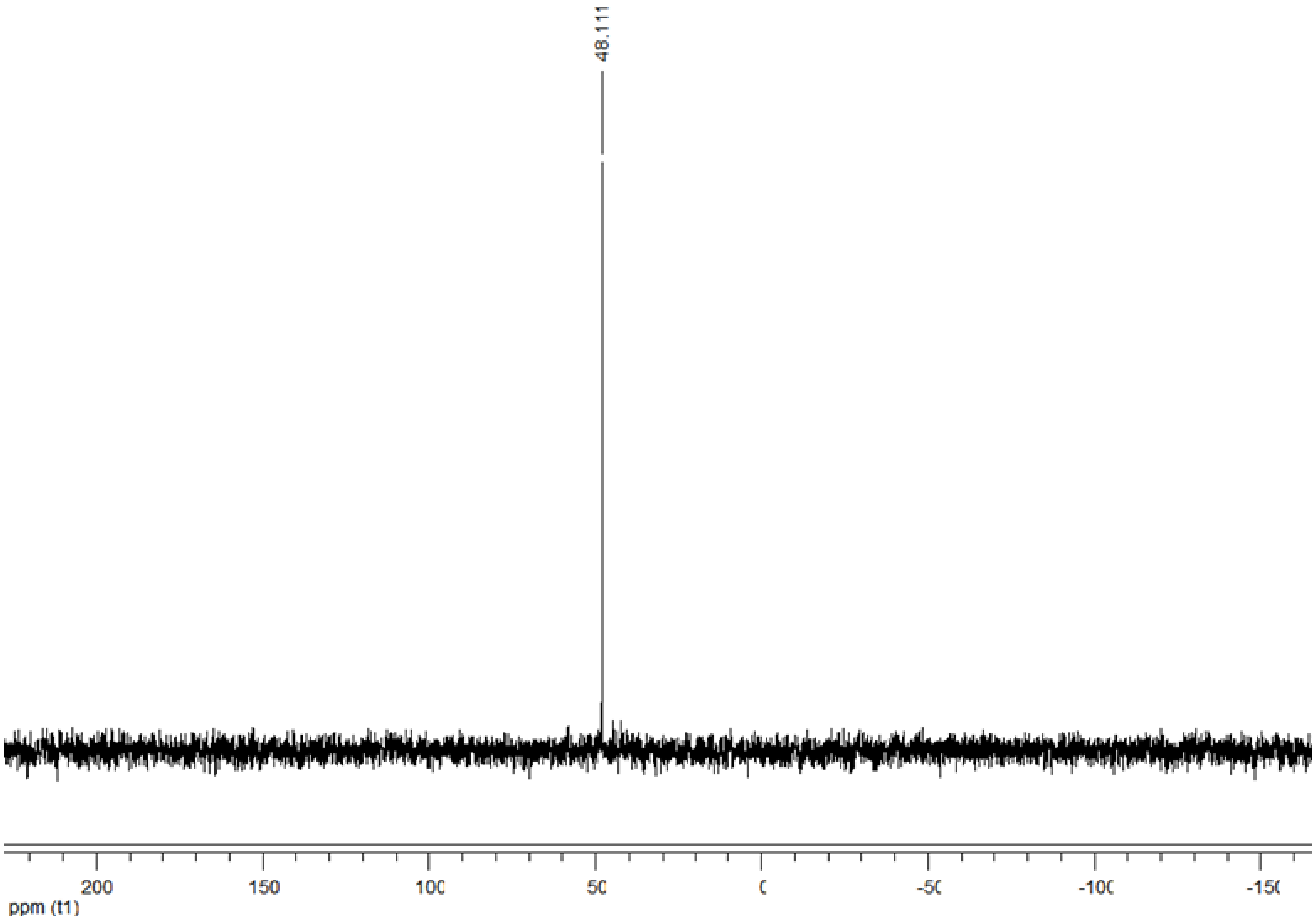

Fig.19. ^{31}P NMR spectra of the hexyl(di-*tert*-butyl)ferrocenylphosphonium tetrafluoroborate (**5c**)

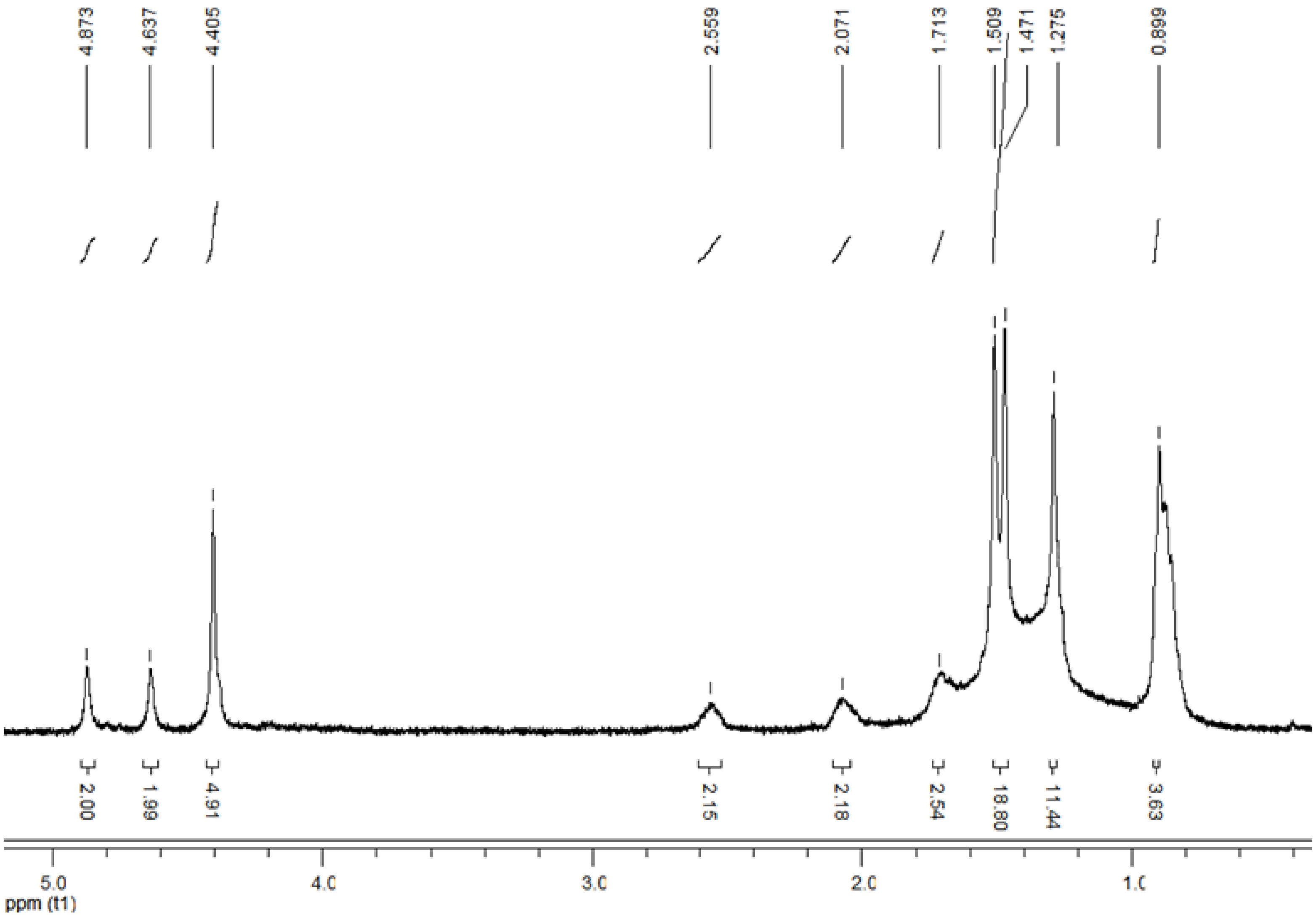

Fig.20. ^{1}H NMR spectra of the decyl(di-*tert*-butyl)ferrocenylphosphonium tetrafluoroborate (**5d**)

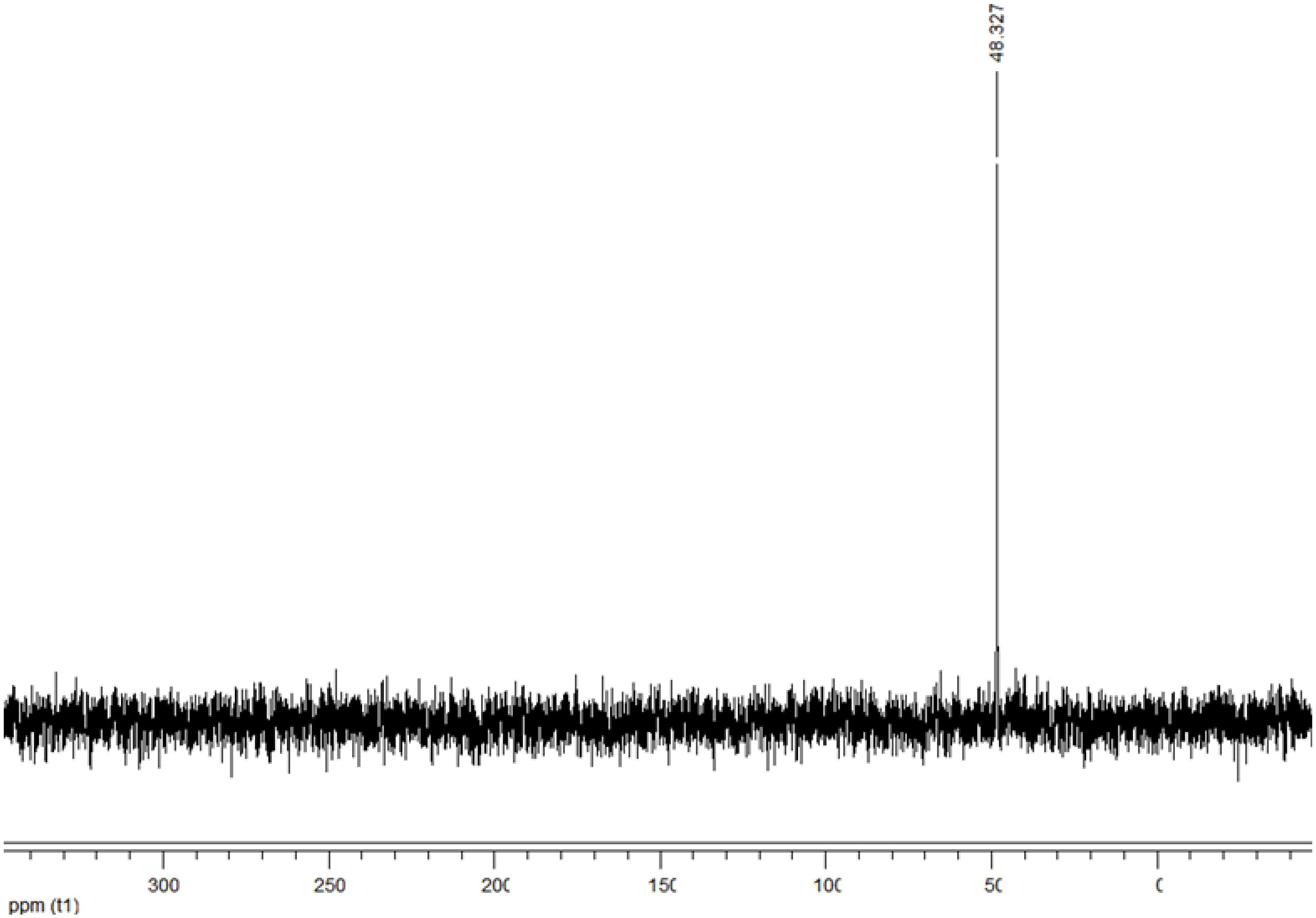

Fig.21. ^{31}P NMR spectra of the decyl(di-*tert*-butyl)ferrocenylphosphonium tetrafluoroborate (**5d**)

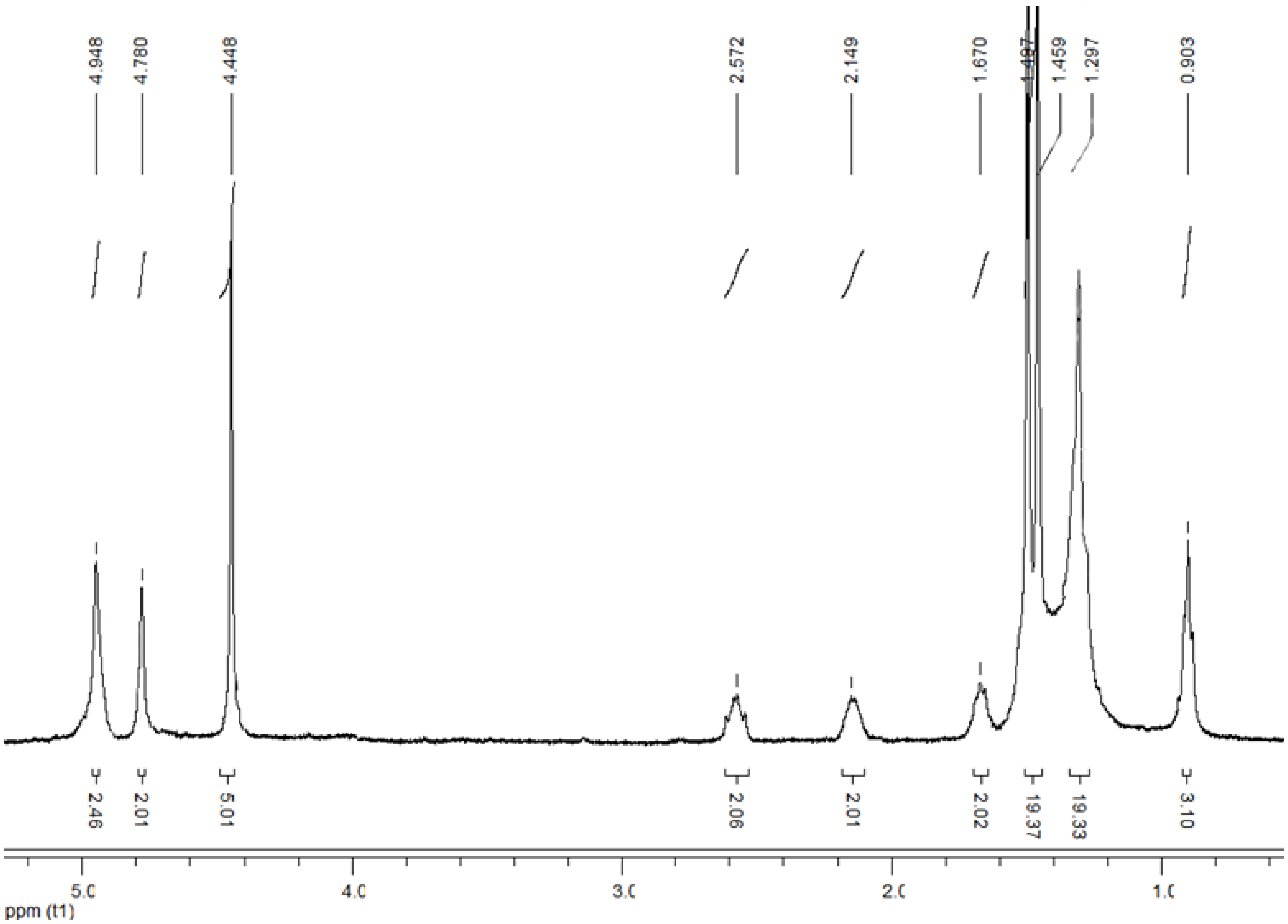

Fig.22. ^{1}H NMR spectra of the tetradecyl(di-*tert*-butyl)ferrocenylphosphonium tetrafluoroborate (**5e**)

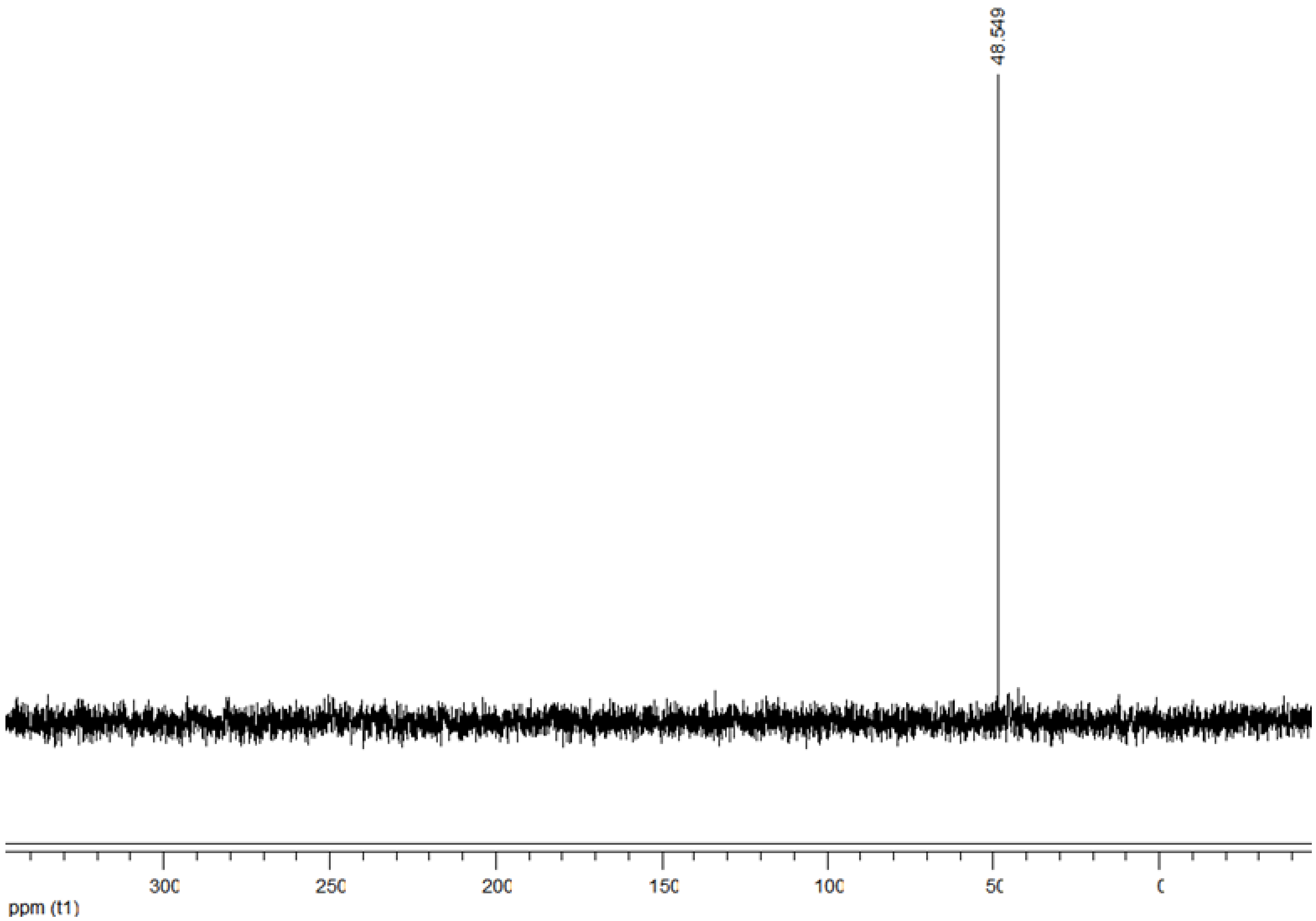

Fig.23. ^{31}P NMR spectra of the tetradecyl(di-*tert*-butyl)ferrocenylphosphonium tetrafluoroborate (**5e**)

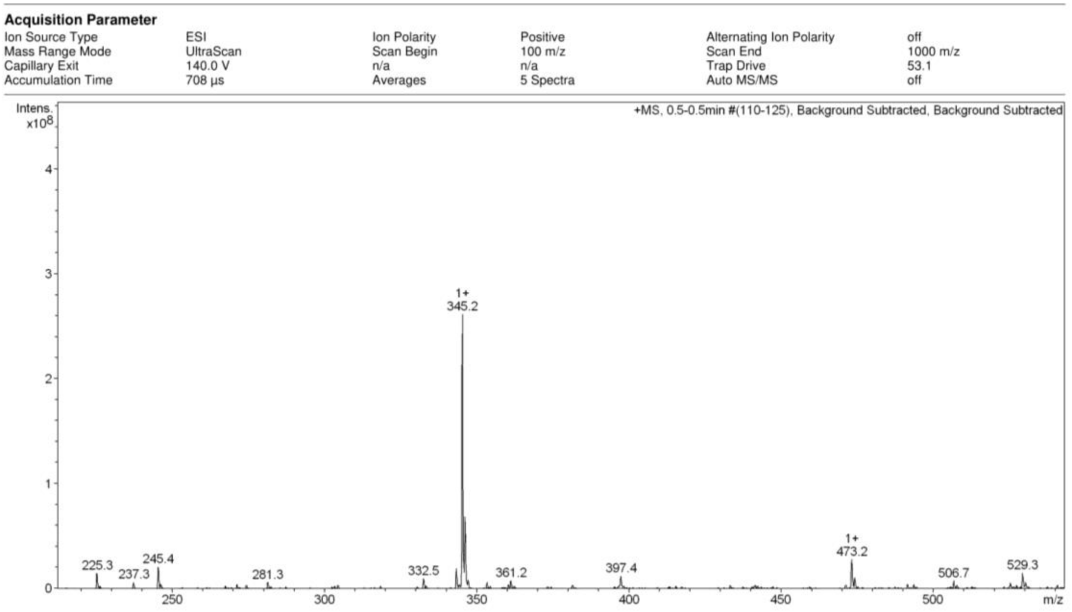

Fig.24. ESI-MS spectra of the methyl(di-*tert*-butyl)ferrocenylphosphonium bromide (**4a**)

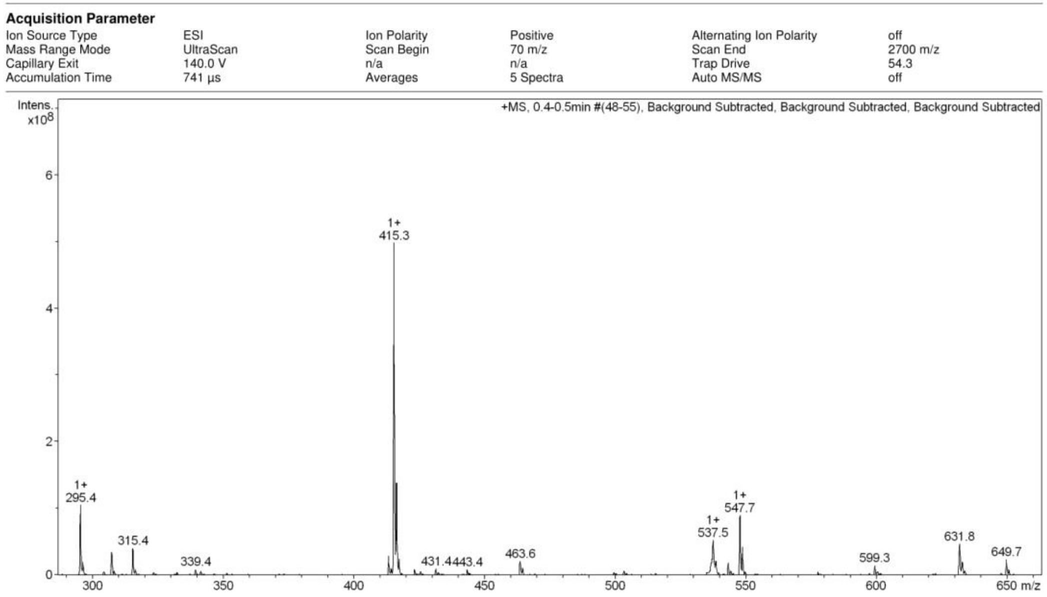

Fig.25. ESI-MS spectra of the hexyl(di-*tert*-butyl)ferrocenylphosphonium bromide (**4c**)

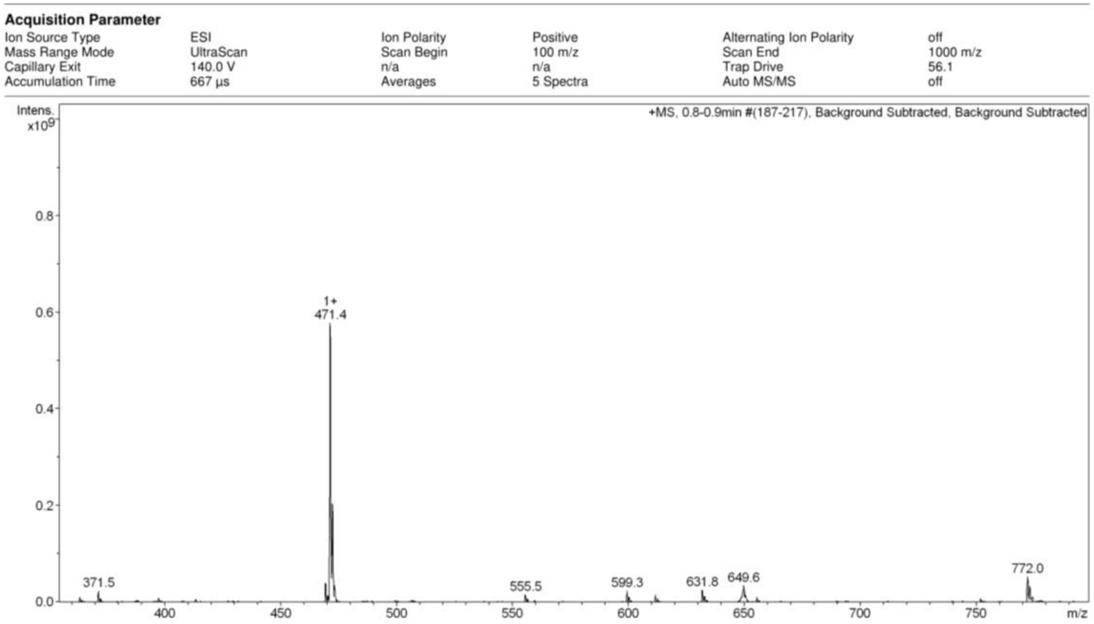

Fig.26. ESI-MS spectra of the decyl(di-*tert*-butyl)ferrocenylphosphonium bromide (**4d**)

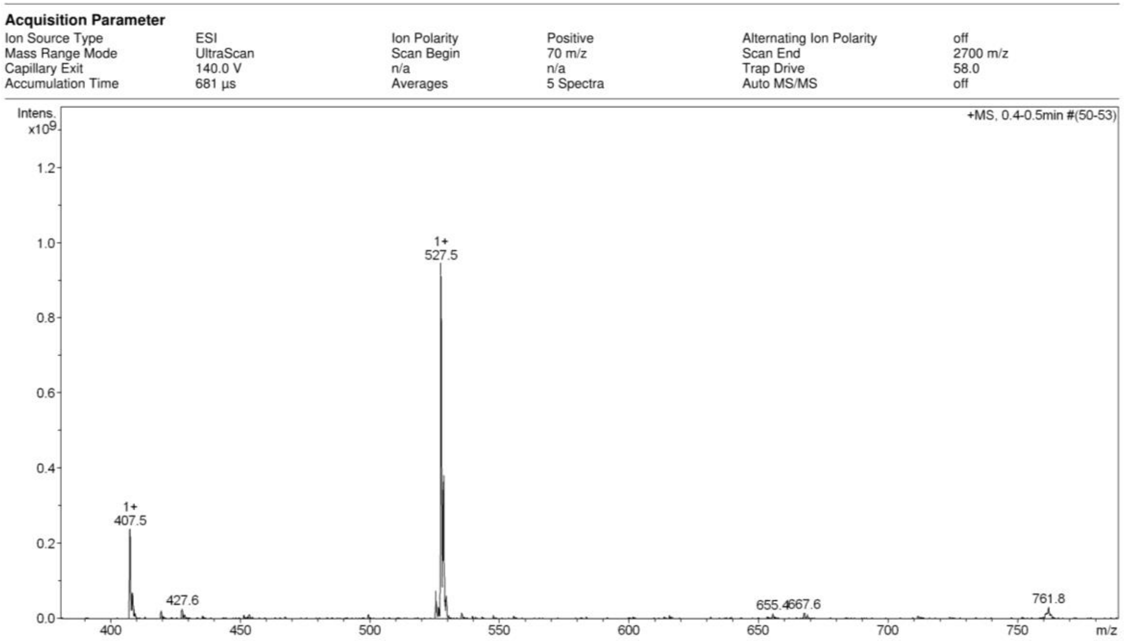

Fig.27. ESI-MS spectra of the tetradecyl(di-*tert*-butyl)ferrocenylphosphonium bromide (**4e**)

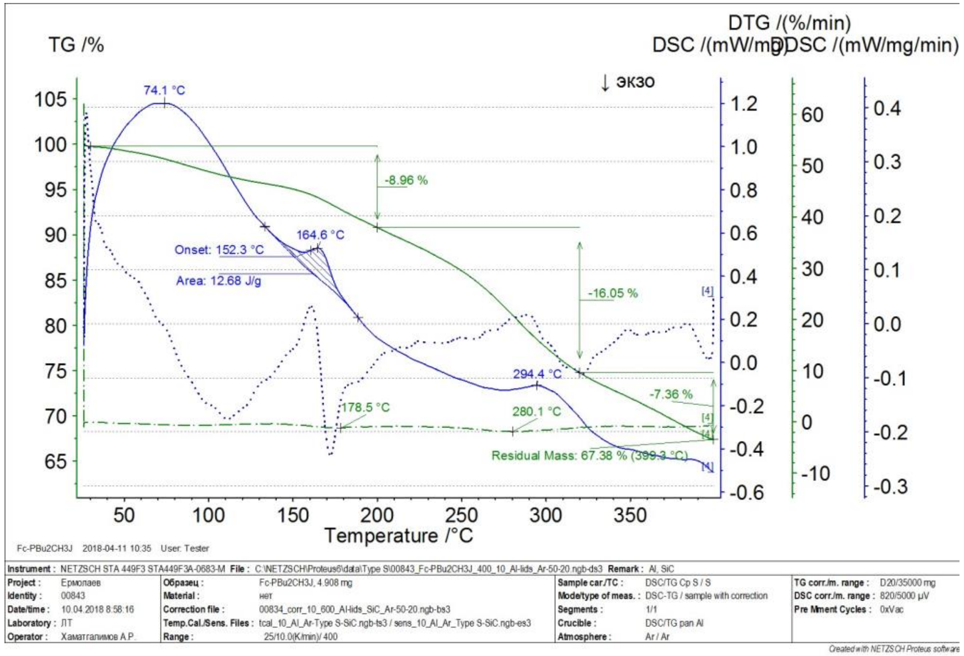

Fig.28. TG-DSC curve of the methyl(di-*tert*-butyl)ferrocenylphosphonium iodide (**4a**)

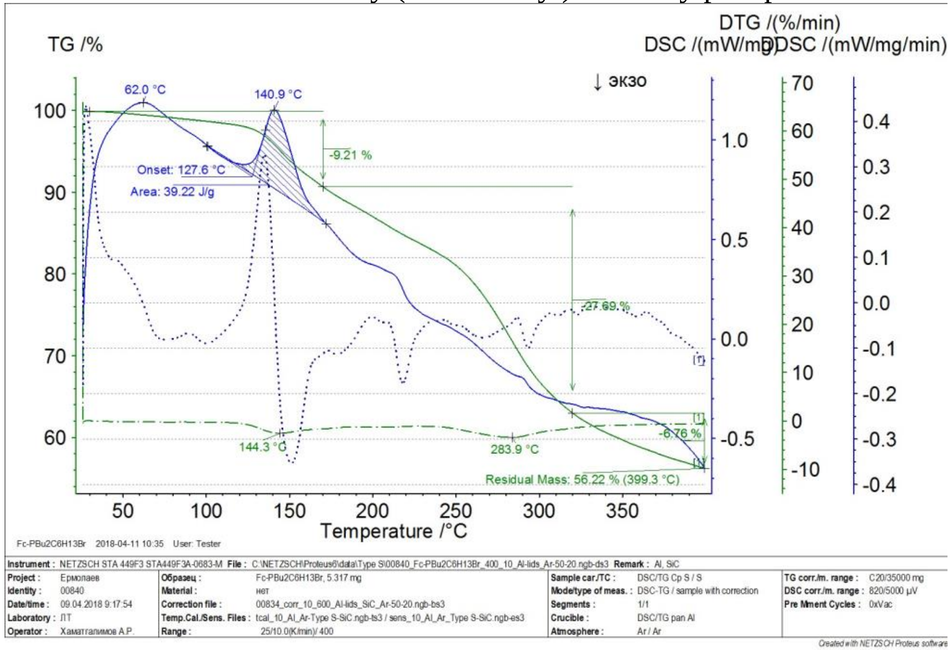

Fig.29. TG-DSC curve of the hexyl(di-*tert*-butyl)ferrocenylphosphonium bromide (**4c**)

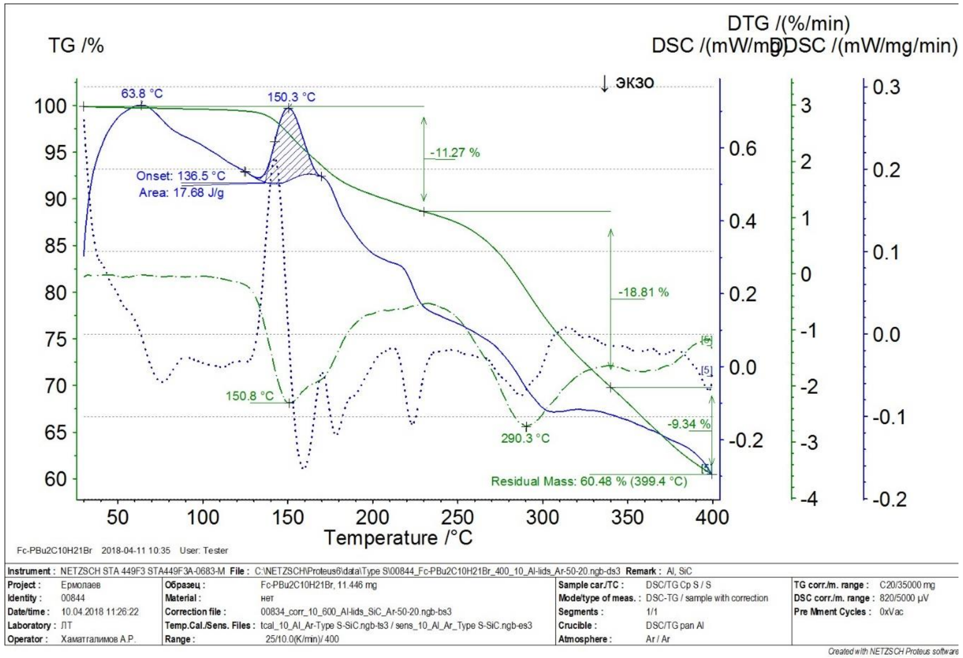

Fig.30. TG-DSC curve of the decyl(di-*tert*-butyl)ferrocenylphosphonium bromide (**4d**)

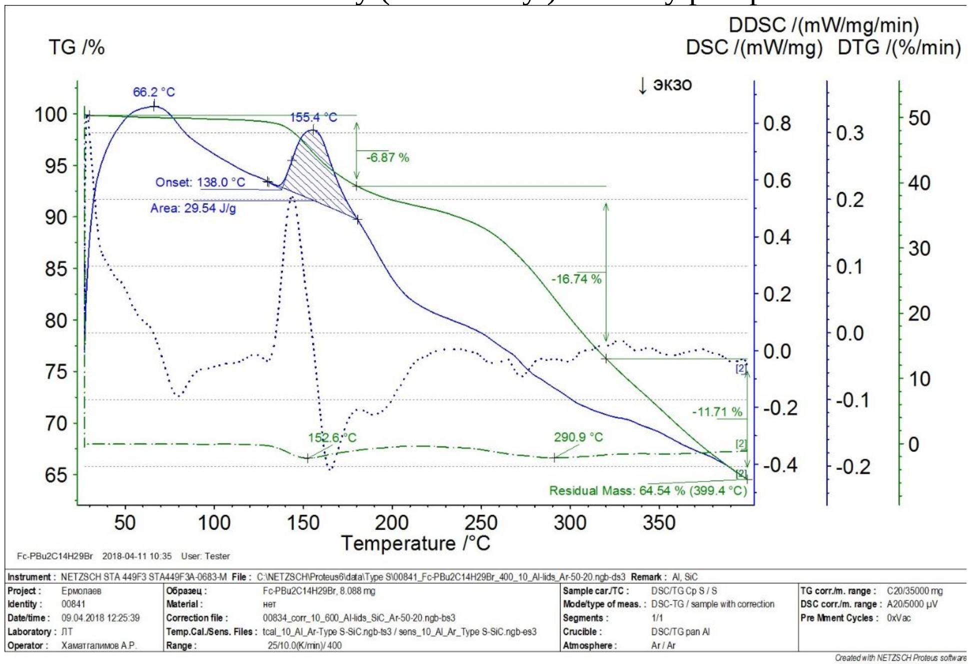

Fig.31. TG-DSC curve of the tetradecyl(di-*tert*-butyl)ferrocenylphosphonium bromide (**4e**)

Acquisition Parameter

Ion Source Type	ESI	Ion Polarity	Positive	Alternating Ion Polarity	off
Mass Range Mode	UltraScan	Scan Begin	70 m/z	Scan End	2700 m/z
Capillary Exit	140.0 V	n/a	n/a	Trap Drive	54.3
Accumulation Time	286 µs	Averages	5 Spectra	Auto MS/MS	off

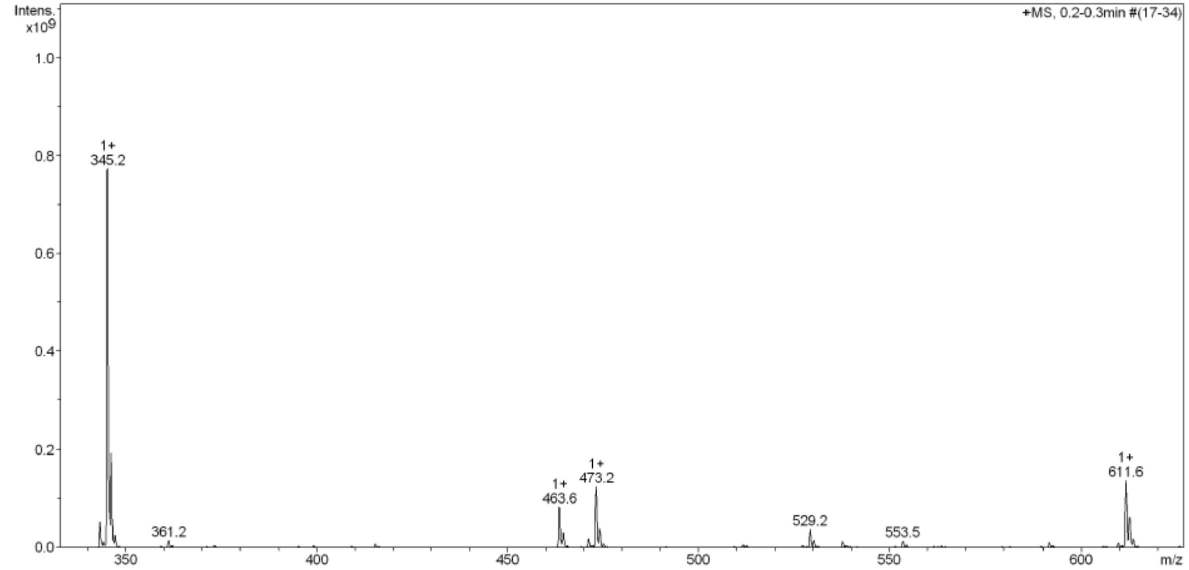

Fig.32. ESI-MS spectra of the methyl(di-*tert*-butyl)ferrocenylphosphonium tetraflouroborate (**5a**)

Acquisition Parameter

Ion Source Type	ESI	Ion Polarity	Negative	Alternating Ion Polarity	off
Mass Range Mode	UltraScan	Scan Begin	70 m/z	Scan End	2700 m/z
Capillary Exit	-140.0 V	n/a	n/a	Trap Drive	48.4
Accumulation Time	4503 µs	Averages	5 Spectra	Auto MS/MS	off

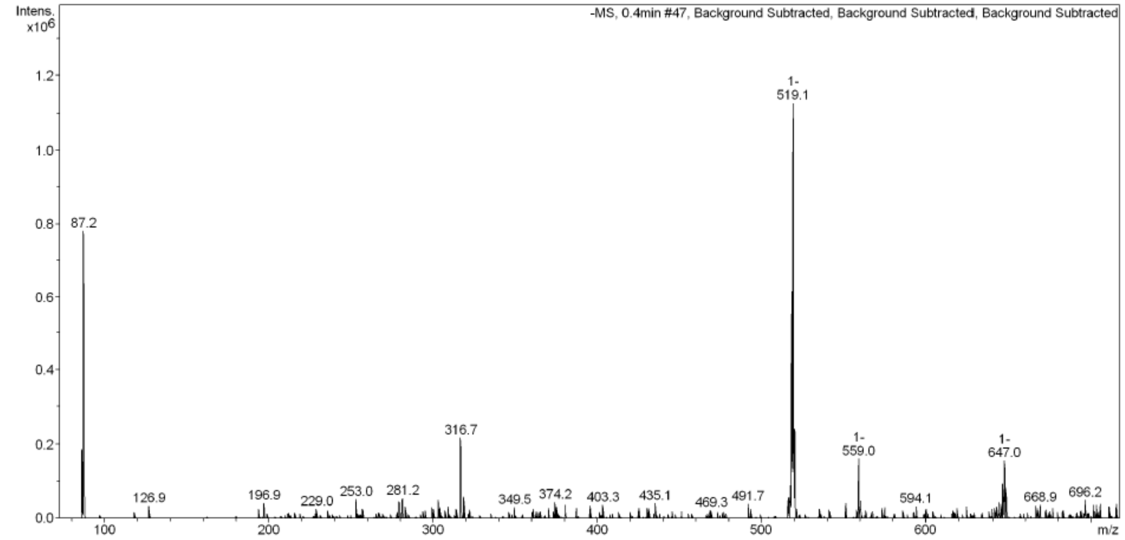

Fig.33. ESI-MS spectra of the methyl(di-*tert*-butyl)ferrocenylphosphonium tetraflouroborate (**5a**)

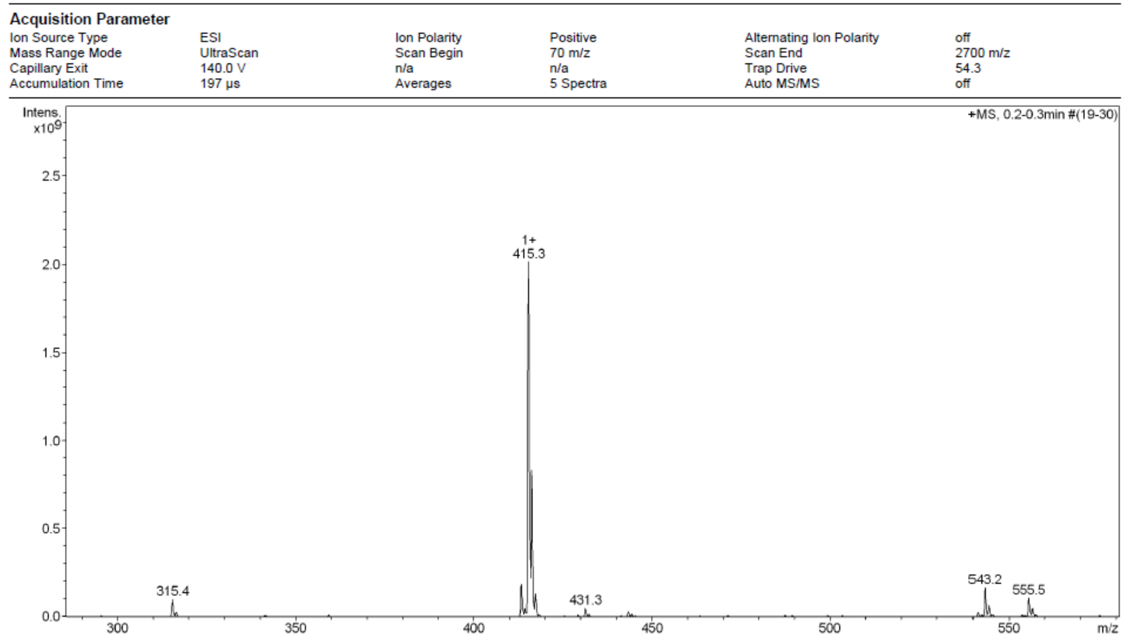

Fig.34. ESI-MS spectra of the hexyl(di-*tert*-butyl)ferrocenylphosphonium tetraflouroborate (**5c**)

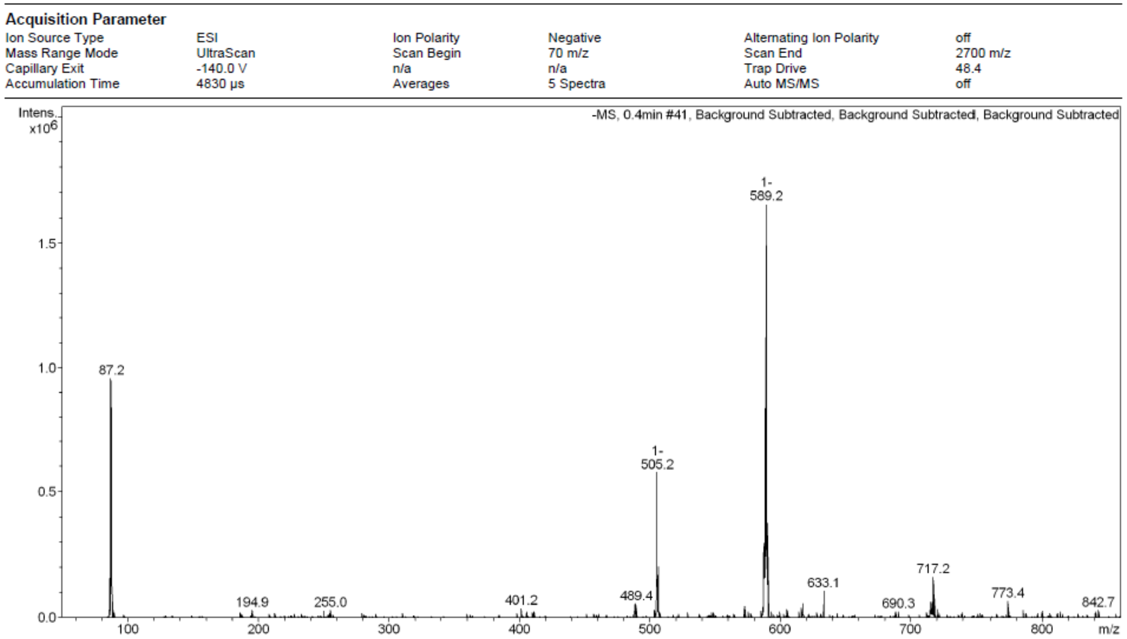

Fig.35. ESI-MS spectra of the hexyl(di-*tert*-butyl)ferrocenylphosphonium tetraflouroborate (**5c**)

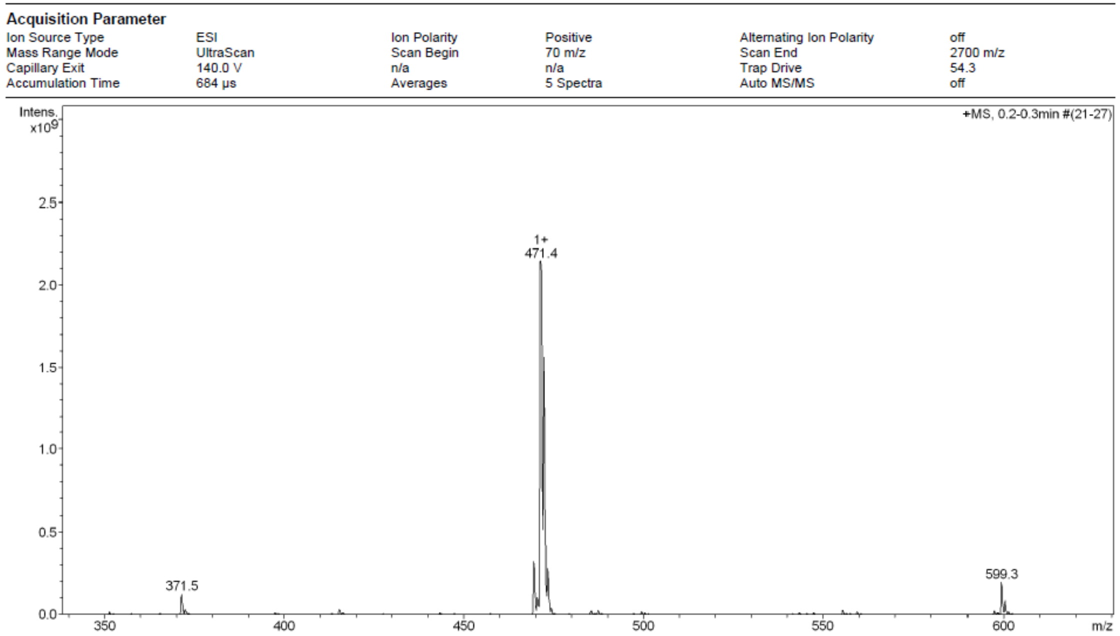

Fig.36. ESI-MS spectra of the decyl(di-*tert*-butyl)ferrocenylphosphonium tetraflouroborate (**5d**)

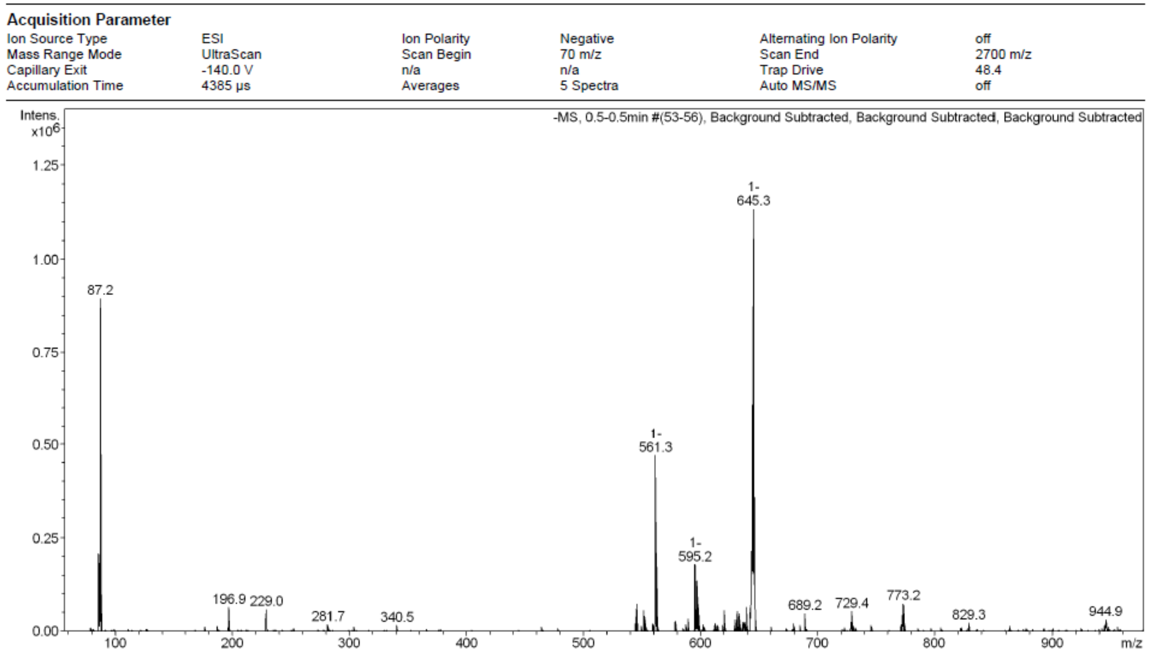

Fig.37. ESI-MS spectra of the decyl(di-*tert*-butyl)ferrocenylphosphonium tetraflouroborate (**5d**)

Acquisition Parameter

Ion Source Type	ESI	Ion Polarity	Positive	Alternating Ion Polarity	off
Mass Range Mode	UltraScan	Scan Begin	70 m/z	Scan End	2700 m/z
Capillary Exit	140.0 V	n/a	n/a	Trap Drive	54.3
Accumulation Time	948 µs	Averages	5 Spectra	Auto MS/MS	off

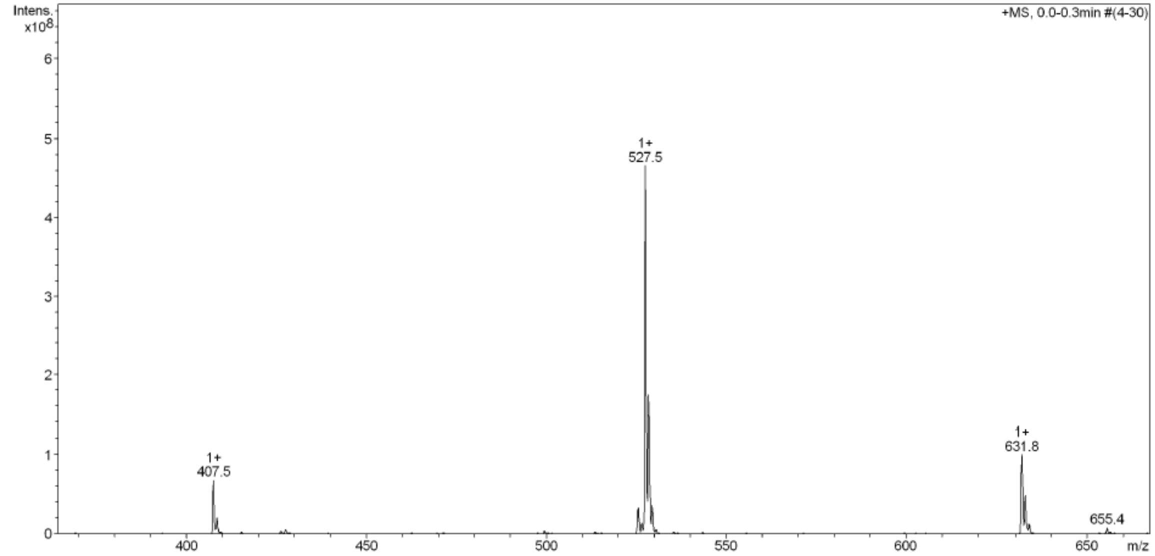

Fig.38. ESI-MS spectra of the tetradecyl(di-*tert*-butyl)ferrocenylphosphonium tetraflouroborate (**5e**)

Acquisition Parameter

Ion Source Type	ESI	Ion Polarity	Negative	Alternating Ion Polarity	off
Mass Range Mode	UltraScan	Scan Begin	70 m/z	Scan End	2700 m/z
Capillary Exit	-140.0 V	n/a	n/a	Trap Drive	48.4
Accumulation Time	3555 µs	Averages	5 Spectra	Auto MS/MS	off

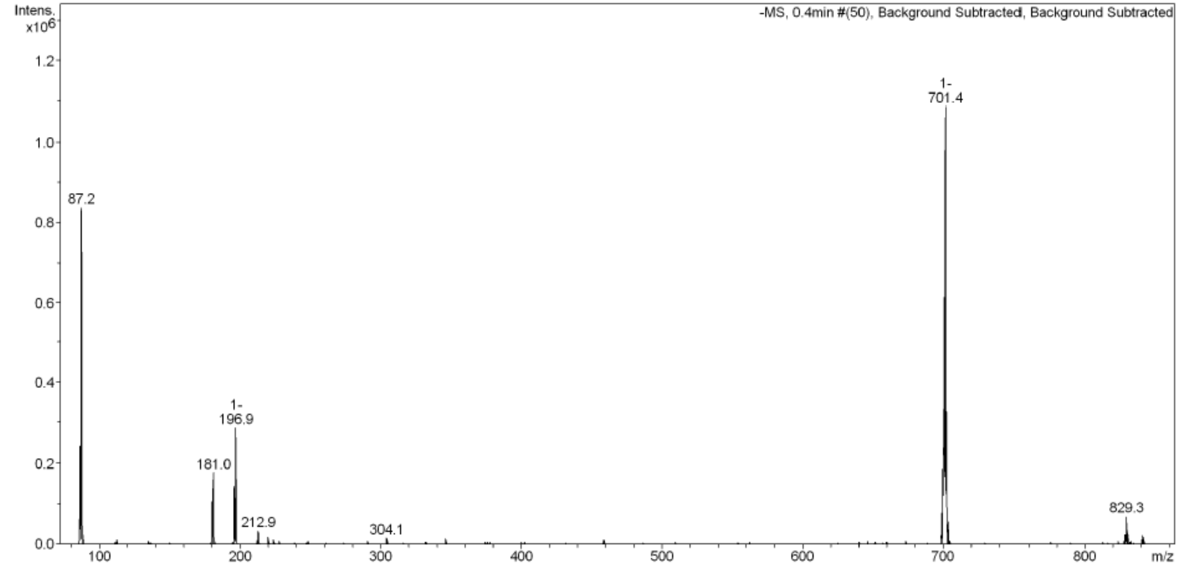

Fig.39. ESI-MS spectra of the tetradecyl(di-*tert*-butyl)ferrocenylphosphonium tetraflouroborate (**5e**)

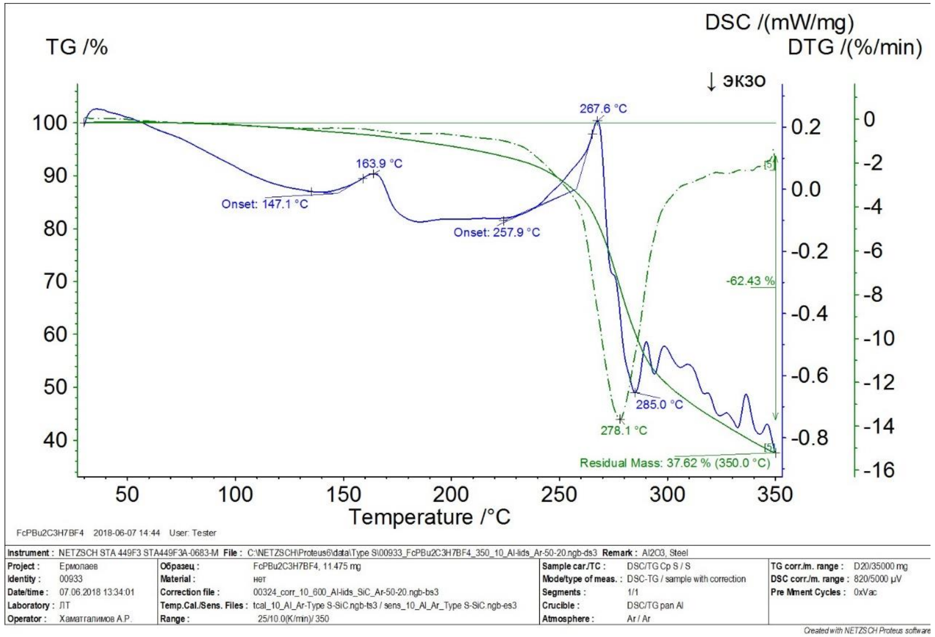

Fig.40. TG-DSC curve of the propyl(di-*tert*-butyl)ferrocenylphosphonium tetrafluoroborate (**5b**)

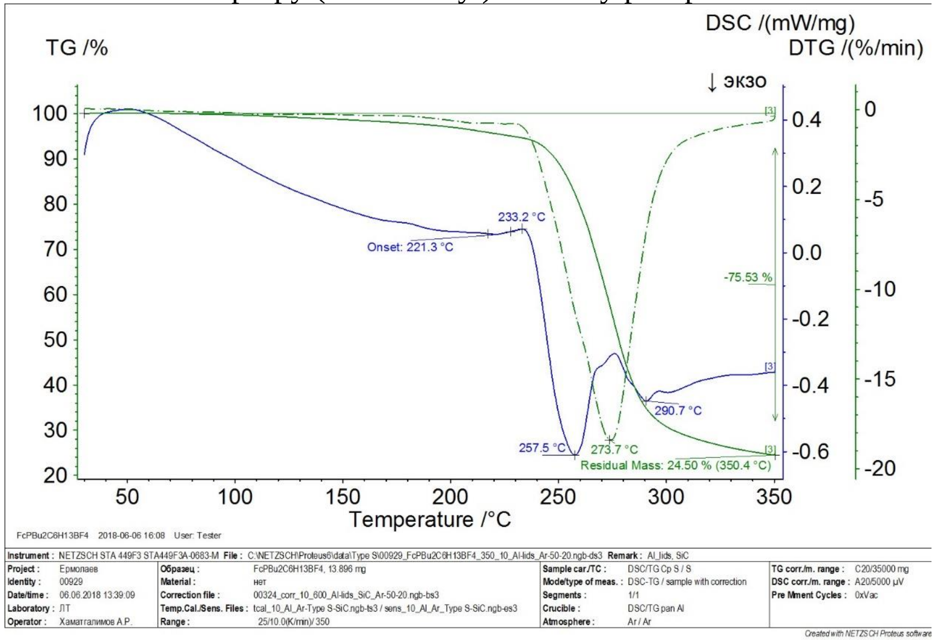

Fig.41. TG-DSC curve of the hexyl(di-*tert*-butyl)ferrocenylphosphonium tetrafluoroborate (**5c**)

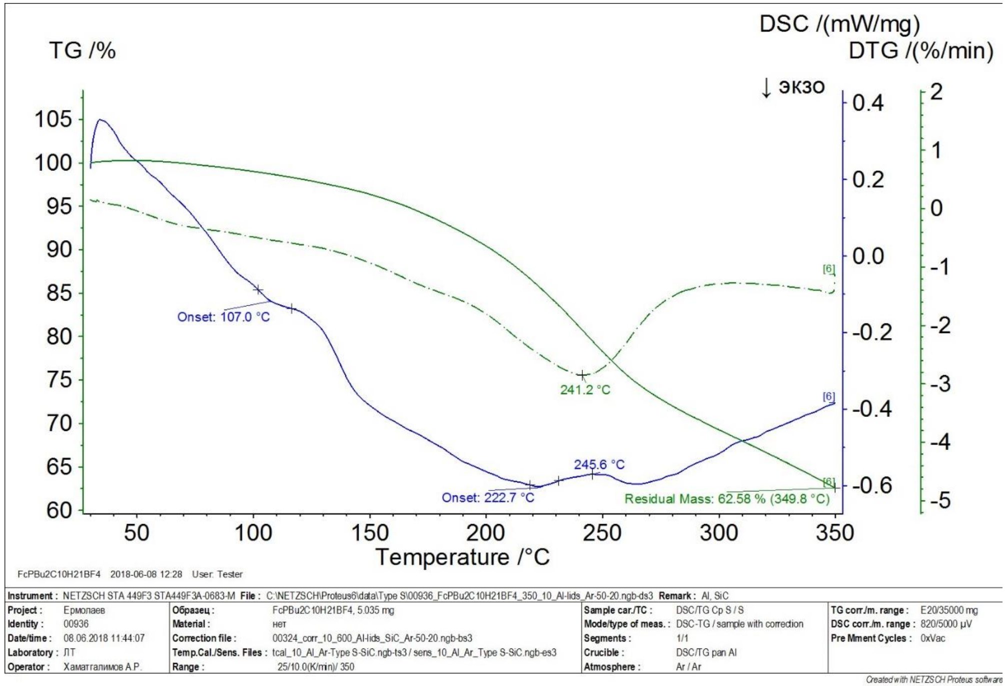

Fig.42. TG-DSC curve of the decyl(di-*tert*-butyl)ferrocenylphosphonium tetrafluoroborate (**5d**)

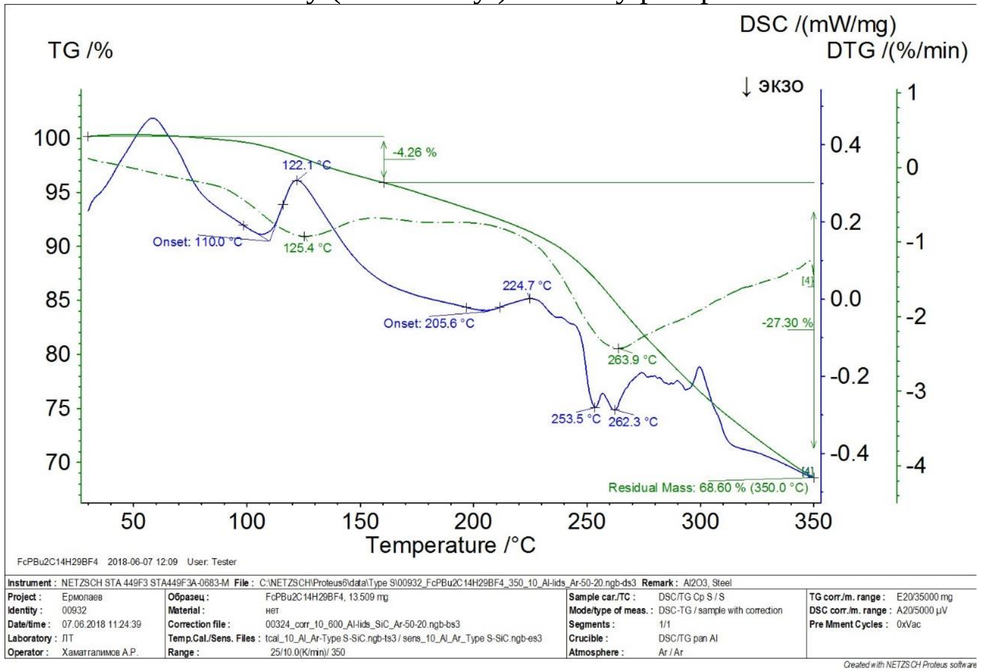

Fig.43. TG-DSC curve of the tetradecyl(di-*tert*-butyl)ferrocenylphosphonium tetrafluoroborate (**5e**)

[1] Frisch, M. J.; Trucks, G. W.; Schlegel, H. B.; Scuseria, G. E.; Robb, M. A.; Cheeseman, J. R.; Scalmani, G.; Barone, V.; Petersson, G. A.; Nakatsuji, H.; et al. Gaussian 09 Revision A .02 Gaussian, Inc., Wallingford CT, 2016. Gaussian, Inc., Wallingford CT 2016.
[2] Adamo, C.; Barone, V. Toward Reliable Density Functional Methods without Adjustable Parameters: The PBE0 Model. J. Chem. Phys. 1999, 110 (13), 6158–6170.
[3] Weigend, F.; Ahlrichs, R. Balanced Basis Sets of Split Valence, Triple Zeta Valence and Quadruple Zeta Valence Quality for H to Rn: Design and Assessment of Accuracy. Phys. Chem. Chem. Phys. 2005, 7 (18), 3297.
[4] Grimme, S.; Antony, J.; Ehrlich, S.; Krieg, H. A Consistent and Accurate Ab Initio Parametrization of Density Functional Dispersion Correction (DFT-D) for the 94 Elements H-Pu. J. Chem. Phys. 2010, 132 (15), 154104–154119.
[5] Becke, A. D.; Johnson, E. R. A Density-Functional Model of the Dispersion Interaction. J. Chem. Phys. 2005, 123 (15), 154101–154109.
[6] Johnson, E. R.; Becke, A. D. A Post-Hartree-Fock Model of Intermolecular Interactions: Inclusion of Higher-Order Corrections. J. Chem. Phys. 2006, 124 (17), 174104–174109.
[7] Grimme, S.; Ehrlich, S.; Goerigk, L. Effect of the Damping Function in Dispersion Corrected Density Functional Theory. J. Comput. Chem. 2011, 32 (7), 1456–1465.
